# Recent Advances in Targeted Nanocarriers for the Management of Triple Negative Breast Cancer

**DOI:** 10.3390/pharmaceutics15010246

**Published:** 2023-01-11

**Authors:** Rajesh Pradhan, Anuradha Dey, Rajeev Taliyan, Anu Puri, Sanskruti Kharavtekar, Sunil Kumar Dubey

**Affiliations:** 1 Department of Pharmacy, Birla Institute of Technology and Science, Pilani 333031, India; 2 Medical Research, R&D Healthcare Division, Emami Ltd., Kolkata 700056, India; 3 RNA Structure and Design Section, RNA Biology Laboratory (RBL), Center for Cancer Research, National Cancer Institute—Frederick, Frederick, MD 21702, USA

**Keywords:** triple-negative breast cancer, biological barriers, tumor microenvironment, nano-therapeutics, inorganic nanoparticles, organic nanoparticles, organic-inorganic hybrid nanoparticle

## Abstract

Triple-negative breast cancer (TNBC) is a life-threatening form of breast cancer which has been found to account for 15% of all the subtypes of breast cancer. Currently available treatments are significantly less effective in TNBC management because of several factors such as poor bioavailability, low specificity, multidrug resistance, poor cellular uptake, and unwanted side effects being the major ones. As a rapidly growing field, nano-therapeutics offers promising alternatives for breast cancer treatment. This platform provides a suitable pathway for crossing biological barriers and allowing sustained systemic circulation time and an improved pharmacokinetic profile of the drug. Apart from this, it also provides an optimized target-specific drug delivery system and improves drug accumulation in tumor cells. This review provides insights into the molecular mechanisms associated with the pathogenesis of TNBC, along with summarizing the conventional therapy and recent advances of different nano-carriers for the management of TNBC.

## 1. Introduction

Triple-negative breast cancer (TNBC) is an aggressive type of heterogenous breast cancer that tests negative for the expression of estrogen, progesterone, and human epidermal growth factor 2 receptors (HER2). These tumors have an aggressive nature, with a tendency for early relapse and metastatic spreading toward the lungs, liver, and central nervous system, as well as a poor prognosis [1,2]. The risk of TNBC is higher in certain ethnic groups, such as Latin, African, and African-American women, as well as women with breast cancer 1 (*BRCA1*) gene mutations. This subtype is most prevalent in young women, and it accounts for 15–20% of all breast cancers diagnosed [3,4]. According to current molecular and morphological studies, invasive ductal carcinomas account for around 90% of TNBC cases, while lobular, apocrine, adenoid cystic, and metaplastic carcinomas account for the rest. Despite sharing the triple negative phenotype, the prognosis of every class is distinct [5]. Further, TNBC is classified into six subtypes (Basal-like 1 & 2, Immunomodulatory, Luminal androgen receptor, Mesenchymal, and Mesenchymal stem-like) based on gene expression profiles [6]. Similarly, Burstein et al. proposed a classification for TNBC that included four subtypes: basal-like immune-suppressed, basal-like immune-activated, luminal androgen receptor, and mesenchymal [7]. In the same study, the basal-like immune-activated subtype was found to be associated with a better prognosis, which is consistent with the findings of other studies showing that TNBC with lymphocytic infiltration had a better prognosis [7]. Recently, TNBC’s diversity in terms of gene expression and the variety of genetic events has been studied. The diverse nature of TNBC, which usually results in distinct responses to therapy, implies that it should further be subdivided from a therapeutic perspective. The discovery of specific molecular markers for TNBC subtypes would surely enhance the diagnosis process, as well as aid the development of predictive biomarkers and targeted therapeutics [8,9].

As there is still no ideal treatment option available for TNBC, this review focuses on discussing the pathophysiological aspects of TNBC and the receptors involved, which helps in achieving target-specific drug delivery using a variety of nanotechnologies. The current treatment approach for TNBC is either individual use of anticancer drugs or along with surgery or radiotherapy. The chemotherapeutic approach involves the use of anticancer agents belonging to classes such as anthracyclines, platinum compounds, and taxanes. Although there is an unmet need in the therapeutic field against TNBC, developing a novel drug is a relatively expensive process. Hence, the scientific community has recently become more interested in studying new drug delivery systems in order to enhance the therapeutic potential of currently available drug molecules [10]. Thus, various nanotechnological approaches involving the use of various organic and inorganic nanoparticles are being explored with essential modifications in order to be effective against TNBC. Herein, we give a comprehensive review focusing on novel drug delivery approaches for TNBC. Furthermore, this review also discusses several distinct approaches for treating TNBC, including gene therapy, photodynamic (PDT), and photothermal therapies (PTT). As it is important to understand the pathophysiology of TNBC to develop new therapeutic approaches, this review also includes a detailed discussion of the molecular mechanisms involved in TNBC pathogenesis.

## 2. Pathogenesis of Triple Negative Breast Cancer

**Figure 1:**
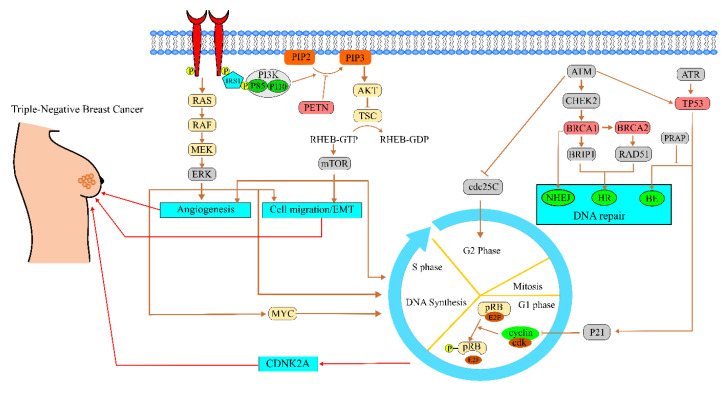
Pathogenesis of TNBC.

TNBC is the most fatal type amongst all breast cancer. It shows the lack of expression on estrogen receptors (ER), progesterone receptors (PR), and HER2 receptor amplification. ER and PR present on the myoepithelial cells and HER2 on the epithelial cells of the breasts are absent in TNBC. The definition of absence here is “less than 1% stain of ER or PR detected by immunohistochemistry and the IHC (immunohistochemistry) score of HER2 to be 0/+1 or +2 with FISH negative’’ [11,12]. One of the significant causes of the development of ER, PR, and HER2 negative cells is the *BRCA* gene mutation. The *BRCA1* gene regulates the DNA damage checkpoints and has a significant role in the DNA repair process. It can convert ER-negative cells to ER-positive cells and is required for the normal development of mammary glands. *BRCA1* and *BRCA2* repair the double-stranded break in DNA by a process known as homologous recombination. Homologous recombination is the process, which is governed by ATM, *BRCA1*, and *BRCA2*. When DNA is damaged, ATM is activated, which phosphorylates *BRCA1*, and the homologous recombination process begins with the help of *BRCA2* and RAD51 [13]. The *BRCA* gene mutation causes the repair of DNA double-strand breaks by mutagenic mechanisms rather than homologous recombination, resulting in genetic instability. This genetic instability leads to the development of cancer [12]. Another reason for the occurrence of TNBC is the transformation of the mammalian target of rapamycin (mTOR) pathway. This transformation makes the disease aggressive and invasive. In TNBC, the phosphoinositide 3 kinase (PI3K)/Akt/mTOR (PAM) is activated, which causes cancer angiogenesis, cell growth, and cell proliferation. Activation of mTOR results in the generation of two complexes, namely mTORC1 and mTORC2. mTORC1 is responsible for stimulating the growth of cells, and mTORC2 mediates AKT phosphorylation [14,15]. Additionally, in tumor cells, there is no control over the cell cycle progression, which permits the cells having faults in DNA to move forward to the subsequent step of the cycle, or alternatively, it causes more genetic diversity. TNBC cells undergo repeated genetic alterations in the tumor suppressor protein (p53) and Rb pathway inactivation, while c-Myc pathways mutations are infrequent. In the cell cycle control process, the Rb pathway in the G1/S checkpoint is triggered by phosphorylation of retinoblastoma through cyclin/CDK complex activation, which leads to the instigation of transcription factor E2F via Myc activation. In contrast, inhibition of CDK2 activity via up-regulation of CDNK2 encourages the inactivation of suppressor gene p53, which blocks the senescence in apoptosis pathways. The amplified expression of CDNK2A and related CDNK2′s complex permits evading the cell-cycle regulatory checks in the Rb pathways and allows progression into the G1/S phase. Hence, it leads to high expression of cellular proliferation and growth. This recurrent genetic alteration causes TNBC [16,17]. The representative pathology has shown in Figure 1.

Earlier, scientists considered basal-like breast cancer and TNBC as the same because they have similar pathological features. With the advancement in genetics, it was discovered that most basal-like cancers are triple-negative, but not all of them possess a triple-negative phenotype. Breast cancers are basal-like if the tumor cells express the genes that are generally expressed by myoepithelial or basal cells, such as CK, CK 5/6, CK14, CK17, fascin, p-cadherin, vimentin, αβ crystallin, and caveolins 1 and 2. Basal-like breast cancers show negative ER, PR, and HER2 receptor expression and express epidermal growth factor receptor (EGFR). According to this concept, all basal-like breast cancers should be categorized as triple-negative. However, TNBCs differ from basal-like cancers with respect to their prognostic characteristics. TNBCs show short disease-free survival as compared to triple-negative non-basal-like cancers. The metastatic spread of triple-negative basal-like cancer appears to be different than that of non-basal-like phenotype. The basal-like phenotype of TNBC metastasizes to the brain and lungs and is less likely to spread to bones and axillary nodes [18,19]. Some of the basal cancers have shown the presence of ER and HER2 receptors. Some TNBCs were tested to be negative for basal-like markers such as CK5/6, CK14, and EGFR. Hence, we can conclude that not all basal-like cancers are triple-negative, nor all TNBCs are basal-like [20].

## 3. Conventional Approaches for TNBC

In the current era, the medical field is engrossed with TNBC due to its aggressiveness [21]. The treatment of tumors has progressed from surgery to the use of X-rays. The selection of the treatment for TNBC and its advancement depends on the origin of cancer development, cancer type, and molecular site, as well as the stage of progression [9]. In the late twentieth century, for the management of TNBC, various conventional therapeutic options, including cytotoxic agents and chemotherapy emerged. Also, research work on targeted cancer therapy, which is based on specific molecular targets in neoplastic processes for the betterment of TNBC, is going on. On the other hand, the biotechnology field and PDT have also offered their contributions as innovative developments in clinical oncology [3]. The most commonly used treatment approaches such as chemotherapy, surgery, and radiotherapy are discussed in detail below.

### 3.1. Chemotherapy

Chemotherapeutic drugs are well-established therapeutic approaches for various cancer treatments. It works biologically by interfering with cellular pathways, stopping tumor progression by impairing tumor cells’ ability to proliferate, resulting in apoptosis. This therapy includes alkylating agents, antimetabolites, mitotic inhibitors, and topoisomerase inhibitors [22,23]. Surprisingly, it has been found that TNBC had an excellent response to chemotherapy as compared to other breast cancer subtypes. It was reported that only one-third of patients who had undergone anthracycline or a combination of anthracyclines and taxane chemotherapy showed a complete pathological response due to chemoresistance [24]. Anthracycline and taxanes were found to be substrates of the multidrug resistance 1 transporter, which is the Pgp transporter. This transporter is majorly involved in the efflux of these drugs, decreasing their effectiveness [22]. Further, non-specific targeting, toxicity, poor solubility, and low bioavailability of anti-cancer drugs restrict the use of conventional therapies. Chemotherapy causes several unwanted side effects, such as nausea, alopecia, vomiting, myelosuppression, and fatigue [25]. Chemotherapy causes a number of other side effects that are both stressful and can be fatal for patients. Furthermore, there are high chances of the development of oral and or gastrointestinal mucositis, leading to ulcerations pain anorexia, weight loss, anemia, fatigue, and chances of sepsis formation. In another retrospective study, individual patient data from four different randomized clinical trials were studied for the presence of hematological non-hematological side effects of anthracycline involving chemotherapy. It was observed that hematological toxicities were common, but the incidence increased with age. It was also observed that taxane involving chemotherapy showed increased incidences of sensory neuropathy of grades 3–4. Mucositis grades 3–4 was more common in the case of elderly patients. The study concluded that the dose-intensified regimes showcased increased toxicities and the incidence of hematological toxicities was high in elderly patients after the use of neoadjuvant chemotherapy regimes against breast cancer. 

Additionally, chemotherapy is primarily utilized in combination with surgery to treat different cancer types. Chemotherapy-based drugs are often administered to patients before or after surgery to improve the therapeutic effectiveness. Based on the therapeutic utilization, it is classified into two types i.e., Adjuvant therapy, after surgery and Neoadjuvant therapy i.e., before surgery [26]. A brief discussion related to adjuvant and neoadjuvant chemotherapy has been mentioned below.

#### 3.1.1. Adjuvant Chemotherapy

Adjuvant therapies enhance both overall and disease-free survival. It prevents the risk of metastasis and tumor recurrence activity. Several research findings have reported that taxanes at various doses have shown good efficacy against metastatic breast cancer (MBC). On the other hand, treatment with taxane has not shown significant results for TNBC. Thus, combination based therapy has gained attention towards the management of TNBC. But, it has also found that women with TNBC mostly do not show any improvement after undergoing this therapy as combination approaches of anthracyclines and taxanes show drug resistance [27]. Further, based on combining anthracyclines and taxane, many clinical trials have been conducted with various chemotherapeutic drugs, such as capecitabine plus taxotere, ixabepilone plus capecitabine [15]. It was observed that the efficacy of the ixabepilone and capecitabine combination showed improvement in progression-free survival. Similarly, a pivotal trial of capecitabine plus taxotere showed that capecitabine improves survival when combined with docetaxel, but there is no significant evidence against TNBC [15,27]. Another trial report on anthracyclines showed the benefit of adjuvant anthracyclines. Patients with more than nine affected lymph nodes were assigned either dose-dense conventional chemotherapy or a rapidly cycled tandem high-dose regimen. The results indicated that young TNBC patients benefited from a quickly cycled tandem approach. It resulted in a 5-year event-free survival rate of 71% in TNBC patients, compared to 26% in TNBC patients receiving dose-dense conventional therapy [22]. Thus, it is essential to conduct more clinical trials on different chemotherapy regimens for the advancement of TNBC treatment.

#### 3.1.2. Neoadjuvant Chemotherapy

Neoadjuvant therapy is cytotoxic chemotherapy that is given before surgery and offers a higher pathological complete response rate in TNBC compared to other types of breast cancer. It is useful in the treatment of tumors ≥2 cm [28]. Individuals who have TNBC responded initially to neoadjuvant therapy. It was estimated that the possibility of relapse was greater in the first 5 years compared to other types of cancer. Its individualization is needed as *BRCA1* mutations are basal, but not all basal cancers exhibit *BRCA1* mutations [15]. It was found that neoadjuvant therapy with taxanes and anthracyclines exhibited a pathologic complete response (pCR) rate of 30%. Several limitations have also been reported, including changes in the stage of cancer, remains of a residual intraductal component after breast-conserving surgery, and overtreatment [29,30]. Furthermore, Telli et al. conducted a phase II study of neoadjuvant therapy of gemcitabine, carboplatin, and iniparib for tumor management, and this approach was found to be effective against TNBC-affected patients [29]. Another study conducted by Gerber et al. reported that the addition of bevacizumab to anthracyclines and taxane enhanced the complete pathological response from 27.9% to 39.3% in TNBC patients [31]. Wu et al. evaluated the response and prognosis of taxanes and anthracyclines as neoadjuvant therapies in TNBC patients. The results indicated that patients suffering from TNBC were more sensitive to neoadjuvant docetaxel and epirubicin chemotherapy [32]. Neoadjuvant therapy with anthracycline and cyclophosphamide was found to show efficacy, but drug resistance remained to be a problem [15]. Another study conducted by Fisher et al. compared neoadjuvant and adjuvant chemotherapy, they selected 385 patients suffering from stage I-III TNBC. Patients were segregated as neoadjuvant with pCR, neoadjuvant without pCR, and adjuvant settings. Fisher’s exact test and analysis of variance were used to compare the data. 17% of patients receiving neoadjuvant therapy reported a pathological complete response. The study suggested neoadjuvant therapy to be more beneficial as compared to adjuvant therapy [33].

### 3.2. Surgery

Surgery is a well-established traditional technique used to treat cancer without any preventable tissue damage. Surgery offers minimal damage to the surrounding tissues over radiation therapy and chemotherapy [21]. There are two types of surgical processes involved in TNBC, i.e., lumpectomy and mastectomy. In the case of lumpectomy, only the part of the breast having cancer along with the surrounding normal tissue was removed. While in mastectomy the entire breast is removed. Studies have shown lumpectomy to give better loco-regional recurrence-free survival (LRRFS), disease-free survival (DFS), and overall survival (OS) compared to mastectomy [34]. It has been reported that the combination of breast-conserving surgery with postoperative radiotherapy improves the overall survival and breast cancer-specific survival (BCSS) compared to total mastectomy TNBC patients [35]. Although surgery is a better option, it also has certain limitations, including post-operative pain, side effects due to heavy medications, and nosocomial infections along with influencing the lifestyle of the patient [21]. 

### 3.3. Radiation Therapy

Generally, after mastectomy or conservation breast surgery (CBS), radiotherapy is given as it can improve loco-regional control in breast cancer [36,37]. In addition to this, reports exhibit *BRCA1* mutation leading to high sensitivity to radiation. It has been reported that removal of occult *BRCA1* deficient tumor foci from the breast and surrounding tissue can occur if CBS is followed by radiotherapy, leading to a decreased locoregional recurrence [22,38]. The associated toxicities and the lack of treatment guides for radiotherapy in TNBC have restricted its use [39,40]. One major limitation of this radiotherapy is the cost involved due to the use of complex machinery and technology. Furthermore, it is also associated with some major off-target side effects.

## 4. Challenges towards Management of TNBC

### 4.1. Biological Barrier

Despite the several advantages offered by nanocarriers, overcoming the physicochemical and biological barriers has remained a concern for researchers. In a meta-analysis study conducted, it was estimated that less than 1% of the nanocarriers are targeted to the high enhanced permeability and retention (EPR) region of the tumor. This is attributed to physiological barriers such as endothelial barriers, cellular barriers, clearance by the mononuclear phagocyte system (MPS), and endosomal escape [41]. These barriers are part of the defense system of our body, which offers protection against foreign substances [42]. The electrostatic interactions, steric interactions, hydrophobic interactions, biological interactions, and solvent interactions with the cell membrane depend on the physicochemical properties of nanoparticles, including size, elasticity, surface charge, shape, pKa, and chemistry of ligand. This, in turn, affects the accumulation of nanocarriers in tumor cells [41,43]. Scale-up, approval by regulatory agencies, and efflux by transporters are other hurdles for nanoparticulate drug delivery. The MPS consists of tissue macrophages, bone marrow progenitors, blood monocytes, and dendritic cells. They play a role in the clearance of the nanocarriers and hence affect their pharmacokinetics and tissue distribution. It affects the efficacy and biological half-life of the nanocarrier. After entering the blood, plasma proteins, apolipoproteins, and immunoglobulins get adsorbed on the nanocarrier, forming protein corona. The process is termed as opsonization. The phagocytes take up these nanocarriers attached to the protein corona, thereby clearing them from the body. It mainly occurs in the liver, lymph nodes, and spleen [44]. PEGylation has been found to be one of the approaches for avoiding the nanocarrier’s uptake by phagocytes. It induces the formation of a hydrating layer composed of ethylene glycol units and water molecules. This prevents its recognition by phagocytes and thereby its uptake. It enhances the circulating life of nanocarriers [45]. In order to reach the tumor site, the nanocarrier has to cross the endothelial layer of blood vessels. The endothelial layer is composed of a glycocalyx and proteoglycan layer, which guides the entry of solutes and macromolecules. The continuous, fenestrated, and discontinuous endothelium acts as a barrier for nanocarrier [42,46].

The endothelial gaps present in tumor blood vessels are not uniform throughout the tissue. This results in the uneven distribution of the nanocarrier in tumor tissue. Perfusion also guides the accumulation of nanocarriers in the tumor tissue. Heterogeneous perfusion also results in the uneven distribution of nanocarriers in tumor tissue. The interstitial tumor matrix forms a barrier for the nanocarriers that do not extravasate into tumor tissue. Decreasing the particle size can be one of the approaches to enhance penetration in the interstitial matrix [41]. Extravasation has also been found to be dependent on the hemodynamics of nanocarriers. However, its effect needs to be investigated further. The next barrier is the tumor microenvironment (TME). Collagen and elastic fibers of proteins and glycosaminoglycans form the interstitial space. In tumor tissues, the collagen content is more as compared to the normal tissues. This makes the ECM extremely rigid and limits the entry of nanocarriers in tumor cells [42]. Interstitial fluid pressure and hypoxic core are some other factors that affect the distribution of nanocarriers in tumor tissue. Further, for the nanocarrier to enter the nucleus and exert their effect, they have to cross the cell membrane. Nanoparticle internalization takes place through the process of endocytosis. Nanocarriers reach the lysosomes if taken up by endosomes and phagosomes related to the phagocytotic and clathrin-mediated endocytotic pathway. Caveolin mediated endocytosis has been found to bypass lysosomes in some instances. Several approaches have been investigated to escape the endosomes, including the coating of polymers or membrane-stabilizing peptides. Caveolin mediated endocytosis is seen in nanocarriers when their surface is functionalized by cholesterol, folic acid, or albumin [45,46]. 

Several studies have revealed that particles of size less than 20 nm significantly bind to lipoproteins, whereas particles above 100 nm were found to have an affinity for complement-related proteins. Particles having a size of more than 200 nm were found to be taken up by the liver and spleen because of complement activation. Further, smaller particles were found to exhibit longer circulation half-life. Further, the shape of the nanocarrier determines cycling time, uptake by macrophages, ability to overcome biological barriers, and targeting effects. Solubility, stability, cytotoxicity, and cellular uptake are determined by surface charge. Elasticity has been found to affect tumor uptake and blood circulation.

### 4.2. Tumor Microenvironment

The tumor cells have several other cells in the neighborhood including immune cells, fibroblasts, lymphocytes, tumor cells, signaling factors, dendritic cells, adipocytes, macrophages, growth factors, tumor vasculature, and proteins that are secreted by these cells. These cells form the tumor microenvironment (TME). The tumor cells alter the components of the TME in such a way that it promotes the growth of the tumor. The cross-talk between the stromal cells and the tumor cells also contribute to the growth and sustenance of the tumor [47]. The TME has been found to govern carcinogenesis, impact the growth and the malignant behavior of mammary cancer cells, and also re-program the surrounding cells [48]. 

Several mechanisms of tumorigenesis and disease progression are being investigated based on the TME. Biomarkers in the stromal environment have been identified and studied to develop new therapies for the treatment of TNBC. The quick progression and poor prognosis of TNBC can be explained by the unique features observed in its TME [48]. Cancer associated fibroblasts (CAF) secrete cytokine and inflammatory mediators which promote the growth and invasiveness of tumor cells. They are also involved in cancer pathogenesis as they promote angiogenesis, extracellular matrix (ECM) remodeling, cause inflammation, and govern differential events in epithelial cells. It has been found that CAF help in TNBC progression by activating TGF-β [47]. It is believed that they originate from bone marrow derived cells in TME, normal fibroblasts which respond to cancer cell signals, and TME of epithelial cells. It is also found to be involved in brain metastases [49]. They produce metalloproteinases which are involved in metastasis and invasion [47]. Tumor-infiltrating lymphocytes mainly consist of T-cells. It includes CD8+ cytotoxic T cells, CD4+ cytotoxic helper cells, and CD4+ regulatory T cells. It is observed that TNBC is characterized by a large amount of tumor-infiltrating lymphocytes (TIL) due to somatic mutations. High TIL has been considered as an indication of complete pathological response in TNBC patients. Cancers exhibiting high TILs are generally ER negative. T-cells regulate the immune response in tumors in the initial stages of cancer. However, their interaction with tumor cells forming Tregs promotes cancer progression, aggressiveness, and development [47,48]. These Treg cells secret TGF-β, and IL-10, suppressing immune function. They were found to suppress contact with cells by expressing cytotoxic T lymphocyte associated antigen 4(CTLA-4), preventing its recognition by tumor cells. Tumor macrophages are involved in metastases, invasion, migration, and poor prognosis. They secrete TGF-β and IL-10 which suppress the immune response. Cancer associated adipocytes have the capability to produce cytokines, hormones, growth factors, and adipokines. It was found that they are involved in promoting tumorigenesis and enhancing the aggressiveness of cancer cells. The extracellular matrix is mainly composed of proteins such as structural proteins, glycoproteins, and proteoglycans. It has been found to be involved in the migration, invasion, and growth of tumor tissue [48]. The major constituent of ECM, that is collagen IV plays an important role in cancer invasion by its degradation [49]. Given the characteristic changes that are associated with the TME of TNBC, exploring its nuances and developing strategies for modulating the same makes TME a promising therapeutic target for treating TNBC whilst ensuring that the challenges associated with are addressed as well. 

## 5. Newly Developed Therapeutic Approaches against TNBC

**Figure 2:**
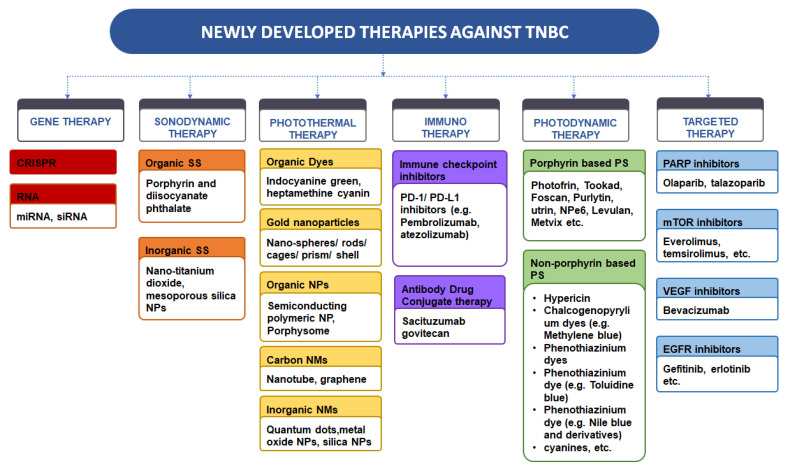
Newly developed therapeutic approaches for TNBC. CRISPR: Clustered Regularly Interspaced Short Palindromic Repeats; EGFR: Epidermal growth factor receptor; miRNA: Micro RNA; mTOR: Mammalian target of rapamycin; NM: Nanomaterials; NPs: Nanoparticles; PARP: Poly (ADP-ribose) polymerases; PD-1/PD-L1: Programmed death ligand 1; PS: Photosensitizer; RNA: Ribonucleic acid; siRNA: Small interfering RNA; SS: Sonodynamic sensitizers; VEGF: Vascular endothelial growth factor.

The management of TNBC is based on its severity and its molecular site. Although various conventional therapeutic approaches are already established for TNBC, there is still an unmet need pertaining to the treatment to be taken as they have several limitations, including non-selective targeting, side effects, and chronic toxicities such as mucositis, thrombocytopenia, and alopecia. Further, the efflux transporters pose a hurdle for anti-cancer drugs. Mainly the ATP binding cassette transporter glycoprotein, which is a P-gp efflux transporter, acts as a significant barrier to several drugs, including taxanes and anthracyclines [50]. The poor prognosis and poor targeting and drug resistance of conventional medicine against TNBC have promoted the interest of scientists in developing new targets and therapies. Since then, newer therapies, such as PDT, vascular endothelial growth factor (VEGF) inhibitors, Gene therapy, PTT, EGRF, etc., have been developed and utilized for effective treatment. These therapies are briefly detailed below, and a pictorial representation of different novel therapies is shown in Figure 2.

### 5.1. VEGF Inhibitor

Vascular endothelial growth factor-A (VEGF) promotes vascular development and angiogenesis in 30–60% of triple-negative breast cancers after binding to VEGF receptor family member 2 (VEGFR) [51,52]. One study reported that mutant p53 along with SWI/SNF, stimulates the expression of VEGFR-2. Thus, mutant p53 can be targeted for breast cancer treatment. Also, JAK2/STAT3 can be targeted, which is recruited by VEGFR-2 to inactivate MYC and SOX2 in breast cancer stem cells [51]. In a recent study, a biocompatible copolymer nanocomplex was developed to deliver VEGF siRNA for TNBC, which inhibited serum degradation, exhibited negligible toxicity, high tumor penetration, high cellular uptake, and high transfection efficiency. In addition, it has significantly inhibited the migration and invasion of TNBC cells [53]. From a recent study, it is found that DEAE (Diethylamino ethyl cellulose)—Dextran coated paclitaxel nanoparticles developed by the solvent evaporation provided the maximum cellular uptake by MDA-MB-231 cells after 1 h. Further, the siRNA prepared by the self-assembly method enhanced the cellular uptake and endosomal escape of nanoparticles and downregulated the intratumoral VEGF protein levels. Also, the strong anti-tumor activity in A-549 xenografts, significant rise in receptor gene expression, and efficiently targeted gene silencing were observed [54]. A study demonstrated that using Bevacizumab monoclonal antibody as a neoadjuvant increased (pCR) while having no effect on DFS or OS in the adjuvant scenario was seen [52].

### 5.2. EGFR Inhibitors

The epidermal growth factor receptor (EGFR) is a tyrosine kinase receptor belonging to the ErbB family that plays a major role in cell proliferation, differentiation, angiogenesis, metastasis, and protection against cell death pathways as well as inhibition of apoptosis [52,55]. Tyrosine kinase inhibitors such as gefitinib and monoclonal antibodies such as cetuximab have been found to target EGFR. The synergistic effect was observed when a combination of gefitinib, docetaxel, and carboplatin was used. Several monoclonal antibodies target these EGFRs by different mechanisms, including ligand-receptor blockade, inhibiting dimerization, and cell survival signaling pathways [56]. The anti-EGFR antibody-drug conjugate for TNBC is more advantageous than the antibody-only and chemotherapy-only treatments. It targets tumor cell proliferation and inhibits DNA repair via cell membrane or nuclear membrane modulation. It is desirable as it does not affect normal tissues, reduces undesirable effects, and delivers small molecules [57]. Another approach is PEGylated nanomedicine like PEG-liposomal doxorubicin, which displays a conditional internalization by PEG engager as it remains in surface contact with TNBC and shows 100-fold more anti-proliferative activity to EGFR+ TNBC [58]. Furthermore, docetaxel-loaded PEG-poly(epsilon-caprolactone) (PCL) nanoparticles exhibit prolonged drug release for up to 30 days, improved dispersibility, and strong anti-proliferative activity against animal models of TNBC and MDA-MB-231 [59].

### 5.3. PARP Inhibitors

PARP is an initiation and damage recognition repair protein for single-strand breaks (SSB) in DNA. Its inhibition results in the accumulation of SSBs, leading to double-strand break (DSB) formation. PARP inhibitors are novel oral anticancer drugs that show promising results in TNBC treatment in *BRCA*-mutated breast cancer [60]. There are many PARP inhibitors available for use, like Olaparib, veliparib, niraparib, rucaparib, and talizumab [61]. Studies have shown that PARP inhibitors’ benefits are transient in patients with *BRCA*-associated TNBC. Further, several research studies have reported that the high-dimensional single-cell profiling of human TNBC showed that macrophages are the most prevalent immune cell type invading *BRCA* susceptibility associated TNBC. PARP inhibitors have been shown to improve both anti- and pro-tumor features of macrophages by reprogramming glucose and lipid metabolism via the sterol regulatory element-binding protein 1 (SREBF1, SREBP1) pathway. Thus, PARP inhibitors combined with antibodies that target the colony-stimulating factor 1 receptor (CSF1R) significantly increased innate and adaptive immune responses [62].

### 5.4. mTOR Inhibitors

As explained in the pathophysiology of TNBC, activation of mTOR is responsible for several functions that promote carcinogenesis. This pathway involves targeting PI3K, AKT, or both PI3K and mTOR [63]. Targeting both mTOR and PI3K results in increased efficacy as well as toxicity. Significant functions of PAM pathways include cell proliferation, survival, metabolism, and migration. Its role in malignant cell transformation has also been investigated. Ipatasertib is a pan-AKT inhibitor targeting a phosphorylated form of AKT. In the preclinical studies, synergy is observed when ipatasertib and paclitaxel were combined. Another small molecular AKT inhibitor capivasertib’s preclinical evaluation has shown activity even in models with alterations in PIK3CA, AKT, and PTEN [64]. Further, Cretella et al. proved the enhanced anti-tumor efficacy of CDK4/6 inhibitors by combining mTOR inhibitors with impaired glucose metabolism. The results indicated superior efficacy in combining palbociclib and mTOR inhibitors. However, preclinical and clinical studies have to be performed to confirm these results [65]. Kim et al. studied the effects of combining ipatasertib and paclitaxel. They conducted a randomized, double-blind, placebo-controlled, phase 2 trial. The results indicated that the combination showed enhanced progression-free survival. The adverse effects observed were manageable. Further clinical trials have to be performed to confirm its safe and effective use in patients with TNBC [66].

### 5.5. Immunotherapy

The aggressive nature of TNBC has led to the development of targeted immunotherapies. Unlike other breast cancer subtypes, TNBC was found to be immunogenic. Hence, researchers started investigating approaches to boost the host’s immune system. The characteristics that make TNBC immunogenic include higher mutational burden, higher quantities of tumor-infiltrating lymphocytes, and higher programmed death-ligand 1 (PD-L1) expression. Higher immunogenic mutations cause tumor cells to produce new antigens. Present immunotherapy approaches for the destruction of tumor cells include cancer vaccines, immune checkpoint blockades such as PD-1/PD-L1 inhibitors, CTLA-4 inhibitors, induction of cytotoxic T-lymphocytes, adoptive cell transfer-based therapy, and modulation of the TME to increase CTL activity [67,68]. 

A novel class of bispecific antibodies targeting CD3-Trop2 (trophoblast cell-surface antigen 2) or CD3-CEACAM5 (carcinoembryonic antigen-related cell adhesion molecule 5) significantly inhibited TNBC cell growth when combined with human PBMCs (peripheral blood mononuclear cells). TNBC cells express bispecific antibodies-Fc fusion proteins that target EGFR, human epidermal growth factor receptor 3 (HER3), receptor tyrosine kinase, human epidermal growth factor receptor 3 (HER3), and Notch. Several targets, such as Trop2, CEACAM5, EphA10, P-cadherin, EpCAM, EGFR, and mesothelin, have also been included in immune cell-redirecting bispecific antibodies, and combination therapy with immune checkpoint inhibitors has been studied. Also, TNBC cells overexpress two receptor tyrosine kinases, Axl and cMet, and blocking both of them with bispecific antibodies helps to target TNBC cells [69]. A study was published that used nanomicelles to target the CD44 mediated apoptosis pathway using a hyaluronic acid (HA) coated biocompatible oligomer containing vitamin E and Styrene Maleic Anhydride (SMA) (HA-SMA-TPGS). The HA-SMA-TPGS was self-assembled and encapsulated with a poorly soluble and potent curcumin analogue (CDF) to produce nanomicelles (NM) with excellent parenteral delivery properties [70].

Moreover, Immune Checkpoint Inhibitors (ICI) is another promising immunotherapy. These are the cell surface membrane proteins such as the programmed cell death-1 (PD-1) receptor that are expressed on T-cells. It belongs to the B7 family of checkpoints. Tumor cells express PD-L1, which attaches to the PD-1 receptor present on T-cells. This inactivates T-cells and hinders tumor destruction caused by T-cells. Researchers have developed several anti-PD-1 antibodies and anti-PDL-1 therapeutic antibodies that disturb the immune regulatory checkpoints and activate anti-tumor immune responses by blocking these receptors and avoiding the inactivation of T-cells [71,72]. The ICIs have provided a new horizon in TNBC treatment. Various ICIs are under clinical trials. For example, anti-CTLA-4 mAbs (Ipilimumab), anti-PD-1 mAbs (atezolizumab, durvalumab, avelumab) have shown excellent initial results. But larger clinical trials are required to establish response rate and determine long term efficiency of ICIs [73]. J.A. Kagihara et al. successfully developed Nab-paclitaxel and atezolizumab for the treatment of PDL-1 positive metastatic TNBC. Food and Drug Administration (FDA) has given approval for this combination, which enhanced the progression-free survival in TNBC patients. However, it was observed that it causes some adverse events, including hypothyroidism and rash. Further investigations confirmed that these adverse events were infrequent and consistent as compared to other atezolizumab trials [74].

CTLA-4, also known as CD152, is widely expressed in CD4+, CD8+, NK, and FOXP3+ cells and regulates the T-cell mediated immune response. B7-1 (CD80) and B7-2 (CD-86) ligands are present on antigen-presenting cells with which CTLA-4 associates and negatively regulates T-cell activation. This suppresses the T-cell dependent immune response. Hence, scientists have developed agents that block CTLA-4 and activate the T-cell-dependent immune response. But it was reported that CTLA-4 inhibitors have more side effects as compared to PD-1 inhibitors. For melanoma, Ipilimumab was the first CTLA-4 inhibitor used, whereas, for breast cancer, Tremelimumab and Ipilimumab are being investigated. Other immune checkpoint targets have also been investigated by researchers, including BTLA, VISTA, TIM3, LAG3, and CD47 [72,75]. 

### 5.6. Gene Therapy

Silencing of genes using microRNAs (miRNAs) and small interfering RNAs (siRNAs) is a rapidly growing approach in cancer treatment [76]. This therapy is used for both diagnostic and therapeutic purposes (theranostics). The gene silencing by siRNAs regulates gene expression through RNA interference, which can be used to treat cancer. The siRNA delivery system can be labeled with imaging agents (for example, dextran-coated superparamagnetic nanosized particles) for non-invasive real-time imaging of siRNA delivery to the tumor using magnetic resonance imaging (MRI). The siRNA-labelled mediated delivery may also help in monitoring and predicting the therapeutic outcome [77]. The delivery of siRNA is a major problem since it is easily broken down by nucleases and its negative charge makes it difficult to localize in cells [76]. Liu et al. formulated siRNA in cationic lipid-assisted PEG-PLA nanoparticles, targeting cyclin-dependent kinase 1 for TNBC treatment. They showed that these nanoparticles promote cell death in c-myc overexpressed TNBC cells by inhibiting CDK-1 expression [78]. Alshaer et al. formulated aptamer guided siRNA nanoparticles targeting CD-44 in triple-negative breast cancer. The core was made up of a siRNA-protamine complex, and the shell had an aptamer ligand for targeting CD-44 cells. The results indicated that the formulation exhibited anti-tumor activity [79]. Tang et al. loaded siRNA into lipid-coated calcium phosphate nanoparticles, which increased the delivery of siRNA to TNBC cells. It was observed that the nanoparticles accumulated in cancer cells because of enhanced permeability and retention effect and the ability of the formulation to target cancer cells [80].

MicroRNAs (miRNAs) play a key role in the genesis and progression of TNBC. Hence, they have the potential to serve as diagnostic biomarkers [81]. Generally, there is a downregulation of miRNAs in tumor cells, but some miRNAs are upregulated [82]. The miRNA558 is overexpressed and the cluster miR-17/92, miR-106b, miR-200 family (miR-200a, miR-200b, and miR-200c), miR-155 and miR-21 are also the highly expressed ones. TNBC with the metastasis to lymph node revealed that six miRNAs, namely iR-125a-5P, miR-579, miR-627, miR-424, miR-101, and let-7g, were expressed in lymph node tissues [15]. One reported study demonstrated that combining Orlistat-loaded nanoparticles with doxorubicin or antisense-miR-21-loaded NPs significantly increased the apoptotic impact when compared to single doxorubicin, antisense-miR-21-loaded NPs, orlistat-loaded NPs, or free orlistat treatment [83].

### 5.7. Photodynamic Therapy

This therapy involves photosensitizers, light activatable molecules, the light of wavelength (near IR light), and oxygen. The therapy consists of two stages: photosensitizer administration and illumination with light. Between these two stages lies the incubation period, which decides the location where the photosensitizer will be released, thereby affecting the efficacy of therapy [84,85] After the administration of the photosensitizer, it is distributed in healthy cells as well as tumor cells. Normal cells eliminate the photosensitizer, whereas the tumor cells accumulate it due to morphological differences between the cells since the tumor cells have impaired vasculature and lymphatic drainage [86]. As a result, it does not allow photosensitizers’ elimination. This therapy causes the destruction of tumor cells by the excited-state photosensitizer’s reaction with triplet-state molecular oxygen present in the body to produce reactive oxygen species (ROS) [87,88]. As the near IR illuminates the photosensitizer, the photon gets absorbed, leading to the formation of an excited singlet stage which is unstable. This unstable state is again stabilized when the photosensitizer returns to the ground state by emitting florescence or through internal conversion by radiating heat. This helps to study the pharmacokinetic distribution of photosensitizers in the body. The excited singlet state of the photosensitizer can also undergo intersystem crossing and destroy tumor tissue by type 1 or type 2 reaction. In a type 1 reaction, electrons or hydrogen are directly abstracted from amino acids or guanine in nucleic acid or NADPH. This leads to the formation of a radical anion of a photosensitizer, which donates its electron to oxygen and produces a superoxide anion radical, restoring the photosensitizer. Type 2 reactions include the transfer of energy to molecular oxygen, resulting in the formation of singlet oxygen, a ROS. The type of reaction that will take place depends on oxygen, substrate concentration, and the type of photosensitizer. However, type 2 reaction is more prevalent as energy transfer occurs at a higher rate than electron abstraction. The superoxide anion radical produced by the type 1 reaction is less reactive than the singlet oxygen produced by the type 2 reaction [89,90].

PDT is a promising therapy for TNBC because of its minimally invasive nature, high accuracy, and precise controllability. Cancers that can be easily assessed by light are treated with photodynamic therapy. They include breast cancer, head and neck cancer, lung cancer, oesophageal, oral, and laryngeal cancer. PDT has been proven to kill tumor cells through three mechanisms. The mechanism that will be followed depends on the localization of the photosensitizer, PS type, and concentration. Direct cell death mechanisms include apoptosis, necrosis, and autophagy, and the other two mechanisms include targeting vascular effects and immune reactions. Apoptosis occurs when the photosensitizer targets the mitochondria. Light-induced damage to mitochondria increases the permeability of the mitochondrial membrane; this, in turn, releases cytochrome c in the cytoplasm, which is necessary to activate the caspase-mediated apoptotic pathway. Cathepsins released after PDT damage to mitochondria causes cleavage of proapoptotic protein Bid to tBid. This then interacts with mitochondria, releasing cytochrome c in the cytoplasm and activating intrinsic apoptosis. This mechanism has been found to be exhibited by NPe6. When the damage caused to the cell is quite high that the apoptotic pathways are destroyed, and the photosensitizer is localized in the plasma membrane, necrosis predominates. This results in the release of intracellular materials outside the cell, inducing inflammation [89,91,92,93].

Autophagy is induced with low PDT damage to the cell. Autophagy is normally a protective mechanism, however when the lysosome is damaged, autophagy’s protective ability is exceeded, and cell death occurs. Negatively charged porphyrins such as phenothiazinium methylene blue and NPe6 were found to localize in the lysosome. Lysosome damage was found to activate apoptosis as well as necrosis. Necroptosis is also induced in some PDT damaged cells due to the imbalance of extracellular and intracellular homeostasis, which depends on the mixed lineage kinase domain like-protein and kinase activities of RIPK 3 and RIPK 1. Blood vessel formation is essential for providing nutrition to cancer cells. Hematoporphyrin has been found to obstruct blood flow. Damage to the tumor vasculature damages the endothelial and subendothelial cells. This causes them to round up, increasing the inter-endothelial cell junctions. These damaged cells release clotting factors such as the von Willebrand factor, resulting in the activation of platelets. Thrombus formation, platelet aggregation, and vessel occlusion are induced by platelet interaction and the released sub-endothelium. They also cause vasoconstriction, decreasing blood flow. This reduced blood flow resulted in hypoxia and decreased nutrition, thereby resulting in tumor destruction. PDT causes damage to cells and induces apoptosis and necrosis; these damaged cells release DAMPs (damage associated membrane proteins). DAMPs include HSP70, calreticulin, ATP, and arachidonic acid. PDT-induced damage also causes the release of inflammatory factors, transcription factors, and heat shock proteins. Tumor antigens bind to the heat shock proteins and interact with toll-like receptors, thereby activating antigen-presenting cells. These antigen-receding cells present the antigens to CD4 helper T-cells, activating cytotoxic CD8+ cells and causing the destruction of tumor cells. The main advantage of PDT induced tumor destruction is that it bypasses the resistance mechanism displayed by the tumor cells [90,93].

Shemesh et al. studied the potential of PDT to kill TNBC cells by using thermosensitive liposomes of indocyanine green (ICG). ICG produces ROS on exposure to a near-IR laser of 808 nm. They concluded that it successfully accumulated in tumor cells. Formulating ICG in thermosensitive liposomes offered several advantages, including stability at physiological temperatures and enhanced permeation [94]. Sun et al. designed a multifunctional cationic porphyrin grafted microbubble loaded with HIF 1 alpha siRNA and delivered by ultrasound targeted PDT to treat TNBC. The siHIF cationic porphyrins were converted to nanoparticles inside the body, which resulted in the targeted accumulation of siRNA and porphyrin in cancer tissue. They concluded that this therapy is effective in TNBC treatment [95].

### 5.8. Photothermal Therapies

PTT is an emerging and highly effective non-invasive treatment therapy for cancer that utilizes the photothermal effect of photothermal agents (PTAs) to destroy cancer cells without causing harm to normal cells. This therapy causes thermal burns or thermal ablations on the tumor upon exposure as it produces heat from absorbed light [96]. The thermal damage caused disrupts the cell membrane and denatures the protein, thereby killing the cancer cells. PTT needs a bio-compatible photothermal agent capable of absorbing near-infrared light that is a NIR source. Several nanoparticles with the ability to absorb NIR light are used in photothermal therapy [97]. PTT has high research value as it exhibits rapid recovery in short treatment time without including a complex operation [96].

In the study reported by Valcourt et al. formulated Polylactic-co-glycolic acid (PLGA) nanoparticles loaded with IR820 for the treatment of TNBC using photothermal therapy which developed biodegradable and polymeric nano particles inducing cell death primarily through apoptosis [98]. A study by Zhao et al. formulated HA-coated gold nanobipyramids to treat TNBC through PTT. They coated the nanoparticles with high and low molecular weight hyaluronic acid, 380 kDa and 102 kDa, respectively. Upon irradiation with 808 nm, it was found that high molecular weight hyaluronic acid showed superior efficacy in targeting overexpressed CD44 cells in TNBC cells [70,99]. Zhang et al. formulated gold nanoparticles targeting the epidermal growth factor receptor for enhancing autophagic cell death in TNBC patients on treatment with photothermal therapy. On treatment with this therapy, there was an increase in autophagic proteins such as beclin-1, p62, and autophagic vesicles [100]. Another study by Wang et al. selectively sensitized malignant cancer cells to PTT by targeting CD44 and reducing HSP72 (heat shock protein 72). They formulated gold nanostar and siRNA to reduce HSP72. This selectively sensitized TNBC cells to hyperthermia. Some of the other advantages shown by the formulation were endosome escape, robust siRNA loading capacity, high hemocompatibility, easy RNA synthesis, and high biocompatibility [101]. Tian et al. developed polydopamine nanoparticles that are loaded with JQ1 to inhibit c-myc and PD-L1 to increase photothermal therapy for the treatment of TNBC. JQ1 was found to decrease the expression of PD-L1 and inhibit the BRD4-c-MYC axis. The synergistic treatment induces the activation of cytotoxic T-lymphocytes, enhancing the immune response [102].

### 5.9. Sonodynamic Therapy 

Sonodynamic therapy (SDT) represents use of ultrasound waves to enhance the activity of chemotherapeutic agents [103]. Ultrasound is a mechanical wave which has frequency equal to or higher than 20 kHz [104]. These high frequency waves are used for improving the efficacy of chemotherapeutic agents. Anticancer drugs like nitrogen mustard, cyclophosphamide, bleomycin, adriamycin, etc. can serve as sonosensitizer. SDT is a method based on the synergistic interaction between ultrasound and a substance known as a “sonosensitizer”. Traditional sonosensitizers have very limited clinical applications due to their low efficiency, low retention in cancer cells, and low tumor selectivity. XiaoHan et al. have developed PEG-IR780@Ce6 for SDT, a new sonosensitizer that inhibits cancer cells as well as suppressing their migration and invasion [105]. The increased efficacy of chemotherapeutic agent by SDT is due to its ability to porate (sonoporation) cell membrane for effective penetration of chemotherapeutic agents [106] or its ability to disperse chemotherapeutic drugs in poorly vascularised tissues in solid tumors [107]. This therapy has been widely used to combat TNBC as the tumor cells of this subtype multiply and progress very fast. Xiaolan Feng et al., proposed a deep-penetrating sonochemistry nanoplatform (Pp18-lipos@SRA737&DOX, PSDL) composed of Pp18 liposomes (Plipo), SRA737 (a CHK1 inhibitor), and doxorubicin (DOX) for the controlled production of reactive oxygen species (ROS) upon ultrasound activation and the resulting intercalation of DOX into the nucleus DNA and induction of cell death [108]. By enclosing manganese-protoporphyrin (MnP) in folic acid-liposomes, Huaqing Chen et al. developed a multifunctional nanosonosensitizer system (FA-MnPs). The nanoparticles of FA-MnPs are characterized by strong depth-responsive SDT as well as a strong immunological response that is mediated by SDT. In the presence of ultrasound radiation, FA-MnPs show great acoustic intensity in mimic tissue up to 8 cm deep, and they also produce significant amounts of singlet oxygen (^1^O_2_). The good depth-responsive SDT of FA-MnPs efficiently slackens the growth of both the surface tumors and the deep lesion in TNBC mouse models [109].

## 6. Emerging Nanotechnology Based Delivery Approaches toward TNBC Treatment

**Figure 3:**
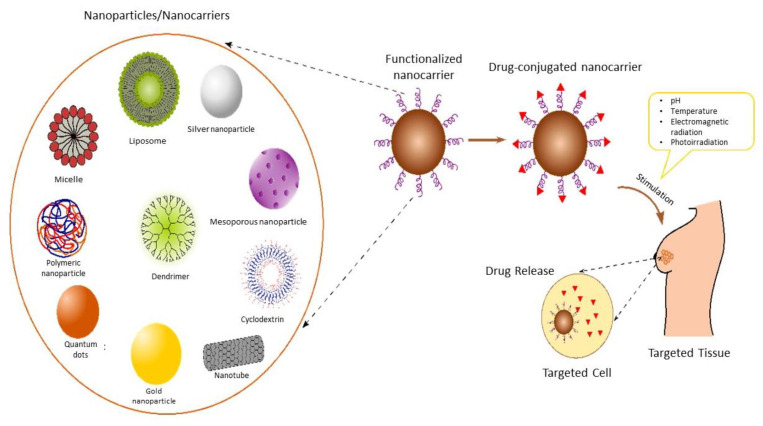
Nanotechnology-based approaches for TNBC treatment.

Nanotechnology is one of the finest tools that has emerged in the past couple of decades, having the potential to fight against severe, difficult-to-manage diseases like cancer. Biomedical sciences have seen the evolution of nanotechnology in targeting cancer, via various approaches in order to have a robust and targeted delivery of diagnostics and therapeutics [110]. Researchers are considering the option of nanomedicine for combinational therapy or as a multidrug delivering system, in case of cancer so as to employ several advantages such as improving the pharmacokinetic profile, decreasing the free drug toxicity, and having a synergistic pharmacological effect of the drugs employed [111]. Other advantages offered by the nano systems are improved therapeutic index, with an increase in localization of the drug in the blood, decreased off-target secondary pharmacological effects, with increased localized targeted action in tumor cells [112,113]. The employment of nanoparticles with primary purpose targeted delivery is based on the characteristic features of the nanoparticles including, average nanoparticulate size, uniformity, surface potential as well as drug loading capacity [112]. The specific advantages offered by nanoparticles can be attributed to the increased large surface area to volume ratio of nanocarriers. Furthermore, the entrapment or loading of drugs in or onto the nanocarriers, has increased the chances of efficient targeting of the tumor cells via the use of single drug molecules or via combination therapy. The nanomedicines are required to be explored more, in targeting the TNBC, as it’s already known that TNBC lacks ER, PR, and HER2 receptors on their membrane. The absence of these receptors makes it difficult for conventional drug systems to target the tumor cells. As a consequence, nanotherapeutics is looked upon as an emerging non-conventional approach in targeting TNBC [114]. One of the basic requirements in the formulation of any nanoparticles is its size, which is desired to be in the range of 1 to 200 nm, modifications in this may alter the dynamical pathway of the particle [115]. The adoption of nano systems in targeting cancer is increased, as it increases the permeation and localization of the drug. Various nanoparticle systems consisting of Liposomes, Micelles, Dendrimers, Solid-lipid Nanoparticles, Polymeric Nanoparticles, Gold Nanoparticles etc. and their details are shown Figure 3.

### 6.1. Organic Nanoparticles

Organic nanoparticles (NPs) are obtained from natural or synthetic organic molecules. The advantage of this nanoparticle includes tailorable synthesis, facile processability, excellent biocompatibility, and low cytotoxicity, revealing outstanding potential in drug delivery, bioimaging, phototherapy, and biomedical application [116,117]. These nanoparticles also offer better advancement including improved drug encapsulation, triggered release, and site-specific targeting. Also, These nanoparticles deliver distinct benefits for toxic drugs such as chemotherapeutics, where off-target toxicity is a key hurdle [118]. These organic nanoparticles are Lipidic nanocarriers, Biological nanocarriers, Polymeric nanocarriers, etc. 

#### 6.1.1. Lipidic Nanocarriers

Lipidic nanocarriers have pronounced advanatages such as they deliver greater availibilty of drug in TME, enhance the biomimetic delivery, achieve reduced side effects, and avoid the development of multidrug resistance (MDR). These nanocarriers can be fabricated from numerous biomaterials including lipid moieties such as fatty acid, phospholipids, lipids etc. These materials can provide several benefits such as being biomimetic, being biodegradable, being more biocompatible, achieving targeted action and improved penetration. These materials are utilised for the development of nanoplatforms such as liposomes, nanostructured lipid carriers (NLCs), Solid lipid nanoparticle (SLNs), and self-micro/nano emulsified drug delivery systems (SMEDDS/SNEDDS), etc. [119].

- Liposomes

Liposomes are the nanoconstructs that can deliver both hydrophobic and hydrophilic drugs. Liposomes were the very first class of nanotherapeutics that got approval for cancer treatment. Liposomes still cover a larger share of nanoparticles in clinical stages [120,121]. These liposomes provide the benefit of biodegradability, biocompatibility, and non-toxic and non-immunogenic nature. On the other hand, the properties of liposomes can be altered considerably by altering the lipid composition, size, and charge on the surface. Liposomes are small spherical-shaped vesicles, which are formulated using cholesterol and natural non-toxic phospholipids. In the case of chemotherapeutics, liposomal formulations enable enhanced efficacy as well as site-specificity in tumor tissues [122,123]. Due to the presence of a phospholipid bilayer, which is amphiphilic in nature, it resembles the mammalian cell membrane, this in return increases the cellular uptake of the same [124]. The liposomal formulations are comparatively low in toxicity parameters as that of drugs alone, and thus improve the drug delivery to the site effectively [125]. Liposomes are being utilized to conjugate many ligands in order to target tumor cells, such as peptides, oligonucleotides, monoclonal antibodies in the case of immunotherapy, and antigen-binding fragments [126]. mAB liposomes which are established and synthesized with optimized procedures offer advantages like cancer specific targeting, high packaging capacity, and prolonged half-life with attached PEG. Y. Si et al. proved that combined standard Gemcitabine and Mertansine which is a polymerization inhibitor led to the formation of mAb-Liposomes against TNBC cells. The inhibition of TNBC cell growth by this combination was observed in cell line xenograft models as well as the patient [127].

- Self-emulsified drug delivery system (SNEDDS/SMEDDS based nanocarrier)

Self-emulsified drug delivery systems serve to make provision for loading a high payload to highly lipophilic drugs via improving its solubility by multiple folds compared to free drug [128,129]. For instance, Guru et al. developed a lipid based self emulsifying system of docetaxol for the management of solid tumors. It has been observed that the carrier provides better retention of drug in biological environment. Also, this study showed greater oral availibilty against marketed formulation (taxotere) [130]. Likewise, Nupur et al. fabricated co-loaded tamoxifen and resveratrol in a SNEDD based nanocarrier for breast cancer treatment. They found that the developed nanocarrier showed enhanced oral bioavailability and improved cytotoxicvity against MCF-7 cell line [131]. In another research report, co-delivery of DOX and LyP-1 in SMEDDS was prepared which reduced the tumor growth and metastasis as well. Also, in-vitro cytotoxicity assays were conducted in p32-expressing BC cells, 4T1 and MDA-MB-231 (TNBC) cell lines and resulted in profound cell killing. LyP-1 is a specific peptide towards the p32 receptor, highly expressed in malignant BC cells [132]. Therefore, self emulsified delivery system is a promising therapeutic approach for the delivery of drug for the management of cancer.

- Solid Lipid Nanoparticle (SLN) and Nanostructured Lipid Carrier (NLC)

Solid lipid nanoparticles (SLNs) and Nanostructured liquid carrier (NLC) are colloidal nanoparticles consisting of an mixture of both liquid lipids and solid lipids which are stabilized by aqueous solution of surfactants or a mixture of surfactants. The lipids utilised for the fabrication of these nanocarriers are many, including triglycerides, fatty acids, steroids, and waxes [119].

Several findings suggest that SLNs can not only uniformly solubilize the hydrophobic drug in lipid matrix system but also provide a drug-enriched shell surrounding the lipidic core. Furthermore, the depostion of drug in the matrix i.e., core type or shell type also plays a crucial role in drug release. For example, drug-enriched SLNs reveal a biphasic drug-release profile; intially nanocarrier show a burst release from the outer shell followed by slow type release pattern release from the lipidic core On the other hand, drug enriched core type provide a long acting sustained release pattern due to enhanced drug diffusional distance from the lipidic core [133]. 

Kothari et al., developed SLN for the delivery of docetaxel –alpha-lipoic acid using various lipids such as GMS, SA, and Compritol ATO 888 for the treatment of TNBC. The finding suggested that the developed carrier exihibited improved cytotoxicity against DTX-SLNs, ALA-SLNs, and free drugs. Additionally, this carrier revealed enhanced apoptosis i.e., 32% with free drug [134]. In another study that was conducted by Pindiprolu et al., they fabricated SLN for the delivery of niclosamine to treat TNBC As per the results, it was found that the drug loaded carrier displayed improved cytotoxicity with enhanced cellular internalization against free drug Furthermore, these SLNs also exhibited increased cellular transfection due to their ability to avoid the efflux pump and hence improve the bioavailability of drugs within the tumor cells [135]. Although, SLN have several advantages over conventional approaches, nonetheless, they have some limitaions as well chances of leakage, less-loading capacity etc. Thus, second generation lipidic nanoparticle i.e., NLC came into existence which offer high drug loading capacity, have decreased risk of gelation, and restricted leakage of the drug upon storage in ccomparison to SLN. Moreover, NLCs also provide a long term exposure of drug towards cancer cell and ultimately, they can enhance the therapeutic efficacy of drug at the tumor site [136]. Sun et al. fabricated NLC based formulation of qurecetin to enhance the oral bioavailability by using soy lecithin, glyceryl tridecanate, glyceryl tripalmitate, Kolliphor HS15. Also, it was shown the improved entrapment efficiency with the prolong -release pattern of quercetin which enhanced cell killing potential against MCF-7 and MDA-MB-231 cells [137]. In another research work by Andey et al., wherein the authors developed lipidic nanocarrier such as liposome, SLN and NLC for the delivery of estrogenic derivative (ESC8). It was observed that lipidic carrier showed good cytotoxicity against standard drug cisplatin. Moreover, SLN showed greater oral bioavailability of ESC8 in comparision with free ESC8 and also showed reduced tumor growth on the xenograft TNBC model [138]. These lipidic nanocarrier showed good pharmacokinetic profile, better therapeutic effectiveness and improved site specific targeting. Therefore, these lipid should fall under GRAS (Generally Recognized as Safe) regulations during the the formrulation development [119].

#### 6.1.2. Micelles

Polymeric micelles are considered an advanced drug delivery system for various therapeutic compounds. Polymeric micelles are advantageous specifically in cases, wherein a poorly water-soluble drug with increased potency as well as toxicity is to be delivered to its targeted site. Polymeric micelles drug delivery technology provides various opportunities for its appropriate use in delivery of the therapeutics. One of the applications is the use of amphiphilic block copolymers as carriers for poorly soluble drugs with the intention of improving the specific targeting as well as the therapeutic potential of the drug. Because of all such advantages, micelles are considered a potential drug delivery system. In one such study, wherein Cetuximab was conjugated with vitamin E forming micelles were used for targeted delivery of docetaxel against TNBC. The formulation of these micelles enhanced the therapeutics effects of docetaxel with the employment of the formulation of Cetuximab conjugated micelles [139]. Iruthayapandi et al. improved the loading efficiency, sustained release, and biocompatibility via the use of protein-based polymeric micelles. Furthermore, in the treatment of TNBC, gelatin-based polymeric micelles hold much importance. Thus, to increase the bioavailability, biodegradability, cellular uptake, increased drug loading, and targeted delivery of poorly soluble drugs, micelles are considered the ideal choice of nano-carriers [140]. Not just the micelles alone, but micelles when combined with the quantum dots along with anti-EGFR and aminoflavone were also employed against TNBC. The conjugated aminoflavone demonstrated enhanced accumulation of the drug at targeted sites of the tumor as compared to encapsulated but non-conjugated aminoflavone. Thus, this resulted in the regression of tumor size in the TNBC xenograft mouse model. The use of such conjugation facilitated hardly any form of systemic toxicity during the treatment period [141]. On the other hand, a few researchers, also designed activated platelets targeting micelles for treating primary and metastatic TNBC. The study included modification of redox-responsive paclitaxel loaded micelles with P-selectin targeting peptide, thus adhering to the surface of the activated platelets and thus targeting circulating tumor cells in the blood, as a result preventing metastasis. Thus, it can also be concluded that the treatment outcomes are improved, as the micelles target tumor-infiltrating platelets against TNBC [142].

#### 6.1.3. Dendrimers

Dendrimers are synthetic, three-dimensional, highly branched macromolecules having one of the dimensions in nanometers. Dendrimers comprise a central core and various functionalities at the periphery [143]. In a detailed manner, the dendrimer has three different parts, namely, a focal core, and building blocks which are repeated units of dendrimers and has multiple functional groups at the peripheral end [144]. Dendrimers are the ideal choice of nanoformulations in case of drugs that have poor solubility in the body’s aqueous environment. Dendrimers as drug delivery vehicles provide characteristic advantages of monodispersity and multivalency [145]. The dendrimers are not only considered for the option of gene delivery but also are associated with gene delivery in the case of TNBC [146]. For instance, Ghosh et al. developed carbon quantum dots from the sweet lemon peel, and conjugated the same with varied generations of polymers including polyamidoamine (PAMAM) in order to form carbon quantum dots-PAMAM conjugates. They have been exploiting these multi-utility dendrimers for diagnostic purposes as well as gene delivery purposes [147]. Dendrimers are garnering a lot of interest due to their use against Polo-like kinase (PLK-1) in TNBC. Dendrimers are modified and complexed in order to deliver the PLK1 siRNA. The dendriplexes formed ultimately lead to an increase in cellular uptake of siPLK1 in MCF-7 and MDA-MB-231 cells. Furthermore, these dendriplexes also enhanced the cell cycle arrest in the sub-G1 phase. Thus, concluding the potential use of phosphorous as well as PAMAM dendrimers in delivering the siPLK1 against TNBC [148]. In another attempt to utilize dendrimers, a doxorubicin-loaded dual-functional drug delivery system was developed by conjugating PAMAM dendrimer, EBP-1, and the cell-penetrating peptide derived from trans-activating transcriptional activator (TAT). This modified drug delivery system was successful in enhancing the anti-proliferative effect of anticancer drugs against MDA-MB-23 cells in vitro, compared to that of the free Doxorubicin effect and convention nano-carrier system. This modification of the drug delivery system not only improved the anti-proliferative activity but also enhanced the drug accumulation at the cancer site in vivo [149]. In another approach for gene delivery through dendrimers, formulations of PAMAM dendrimers were functionalized for delivering the siRNA for TWIST1 gene in TNBC. This silencing was proven efficient because of reduced tumor invasion as well as metastasis [150]. Several other studies substantiate the effectiveness of dendrimers for utilization in cancer therapeutics [151].

#### 6.1.4. Polymeric Nanoparticles

Polymeric nanoparticles have gained tremendous attention over the last decades in terms of their applications in the fields of biology and medicine [152]. Polymeric nanoparticles are mainly colloidal drug delivery systems comprising either natural or synthetic polymers. These have additional advantages over other conventional nanocarriers, in terms of scale-up and manufacturing of the same. Furthermore, these provide the required stability to the systems in the biological fluids [153]. Also because of the absolute pharmacokinetic properties of polymeric nanoparticles, they are considered efficient nanocarriers. [154]. To enhance drug delivery, polymeric engineering of the nanoparticles is one of the highly efficient ways of targeting strategies for anticancer drugs. Silica nanoparticles, loaded with doxorubicin were modified with a polymer, polyethyleneimine, this modified drug delivery system, induced endosomal rupture, and the formulation included HA, binding to CD44 receptors on the cells that are largely expressed in cancer cells. These engineered modifications of the drug delivery system, involving a polymer increased the therapeutic outcome of the delivery system, with a lower dose requirement of the chemotherapeutic agent, thus decreasing the chances of systemic toxicity [155]. In an attempt in using polymersomes, in TNBC, Doxorubicin was encapsulated in the polymersomes which were targeted with hypoxia-responsive peptides, which results in the formation of targeted polymersomes. It was observed that the targeted polymersomes decreased the TNBC cell viability, as well as proved the anti-tumor activity in animal models of TNBC [156]. Furthermore, the therapeutic efficacy of quercetin, a plant-derived flavonoid was enhanced by the development of a nanoparticulate system from D-∝-tocopherol polyethylene glycol 1000 succinate (TPGS) and PLGA and was further evaluated in vitro and in vivo against TNBC. Thus, the study concluded that the use of Quercetin nanoparticles formed via the use of the polymer improved the antitumor and repressed the metastatic effect via inhibiting uPA, thus enabling a newer approach against targeting TNBC [157]. The Polymeric Nanoparticles can also be exploited not only to deliver drugs but also in delivering miRNA and siRNA in association with the chemotherapeutic drug, which would ultimately lead to a reduction in tumor volume and thus its growth. PLGA-b-PEG polymer Nanoparticles delivered both antisense-miR-10b and antisense-miR-21 with a dose 0.15 mg/kg drug dose but when siRNA and Doxorubicin loaded nanoparticles were delivered, the overall tumor size reduction in terms of growth and volume was 8 fold more [158,159]. To form a nanoparticle, a protein polymer, called as elastin-like polypeptide was employed and its surface was coated with FK506 binding protein 12, a cognate receptor for the drug Rapamycin which is a potent drug but having poor solubility. The modification and delivery of the drug through the nanoparticle formed from the polymer enhanced the anti-tumor effect in the TNBC xenograft mouse model [160].

#### 6.1.5. Biological Nanocarrier

Biological nanocarriers are bicompartible and biomimetic type of carrier which consist of various biological cells and its components such as exsosome, platelet, RBCs, nucleic acids, peptides, aptamers, antibodies genes etc. These carriers provide the benefits of mediating better site specific tumor targeting actions and triggering detrimental effects on the tumor cells [161]. Some biological nanocarriers have been discussed below:

- Erythrocyte based nanoparticles

Erythrocytes nanoparticles have risen as one of efficient biocarriers due to its easy method of preparation and high drug loading, biodegradability, and ability to provide a long circulation half-life in the biological environment [162]. Cheng yet al. developed a biotinyl modified cRGD conjugated-RBCs nanoparticle of doxrubucin to treat cancer, wherein, they found accurate targeting, high drug loading, and controlled drug release pattern against free drug. Also, these carriers exhibit better cytotoxicity against MCF-7 cell in comparison to free doxrubucin [163].

- CRISPR nanoparticles

The major drawback in the treatment of breast cancer therapy is drug resistance. The leading reasons for the development of drug resistance are transporter efflux and specific modification in tumor cell genetic composition. Due to this concern, CRISPR based therapy is rising attention towards cancer treatment [164]. Clustered regularly interspaced short palindromic repeats abbreviated as CRISPR is the latest genetic editing tool which upon combination with nanotechnology stands to be a promising therapeutic approach for treating cancers [165]. Zhang et al. fabricated PEG phospholipid-modified cationic lipid nanoparticles (PLNP) for CRISPR/Cas9 to enhance its transfection into the cellular site. In this work, they loaded the Cas9/sgRNA plasmid to obtain core-shell component (PLNP/DNA) which helps in effective transfection of Cas9/sgPLK-1 plasmid into tumor cells. Also the result have shown a subsequent reduction in tumor growth after administration of Cas9/sgPLK-1 plasmid to mouse [166]. Thus, this approach delivers greater efficacy both in-vivo and in-vitro which could be useful for clinical translation against various cancers.

- Exosomes

Exosomes are surface membrane vesicles; chemically they consist of protein and lipid in the body, which are in association with numerous pathological and biological processes. Now a days, exosomes are utilized for the drug delivery as it is a body component which provides benefits such as non-immunogenicity and enhanced circulation time [161]. Tian et al. developed DOX loaded exosomes for effective targeting and site specific delivery to αv integrin-positive BC cells. After administration of DOX loaded exosomes, it was reported that the carrier demonstrated low toxicity with reduced the tumor growth., moreover, this exosome can further undergo surface modification to develop other delivery systems like polymeric, lipidic nanoparticles etc. to obtain site specific targeting [167]. Singh et al. reported that miR-10b (microRNA) loaded exosome could treat metastatic BC cells (MDA-MB-231). These carriers enhance the uptake of miR-10b which help in the reduction of protein expression of associated target genes such as KLF4 and HOXD10 [168]. Several findings suggest that exosomes can modulate the TME and provide a platform for drug delivery to various tumors.

#### 6.1.6. Carbon Nanotubes 

The carbon nanotubes can be used in the delivery and development of therapeutic agents such as peptides, proteins, nucleic acids, genes, vaccines, etc. Carbon Nanotubes can be combined with bioactive peptides, proteins, nucleic acids, and drugs, and thus functionalized for delivering their cargo to cells and organs [169]. Carbon nanotubes are looked upon as the ideal nanocarriers because the functionalization leads to a decrease in systemic toxicity of the nanotubes as well as a decrease in its immunogenic effect. The functionalization also helps in enabling as well as enhancing the dispersion of carbon nanotubes in the biological aqueous based fluids. To enhance the delivering effect of nanotubes, an attempt was made to develop multiwalled carbon nanotubes for carrying and delivering platinum against TNBC. Thus, the results indicated that the functionalized multiwalled carbon nanotubes decreased the cell viability by 40% based on 48 h. exposure. Although, they decreased the caspase-3 and p53 expression, indicating failure to overcome TNBC resistance [170]. Another approach in targeting TNBC is the use of platinum based acridine loaded in carbon nanotubes. With the help of noncovalent πstacking, the platinum acridines are adsorbed over the surface of carbon nanotubes. This bonding and interaction between the carbon nanotubes and platinum acridines ensure the controlled release of high doses of platinum in the TME, thus limiting dose-proportional toxicities. It was observed that these platinum-acridine carbon nanotubes were effective against various models of TNBC, including SUM159, BT20, MDA-MB-468, and MDA-MB-231 [171]. In another study, the researchers combined the multi-walled carbon nanotubes with Hyaluronic acid, and ∝-Tocopherol succinate was loaded with the cytotoxic drug, Doxorubicin against TNBC cells. Specifically, these TNBC cells were overexpressing the CD44 receptors. The study resulted in significant growth repression and increased apoptotic effect when compared with other formulations [172]. Thus, it is observed that carbon nanotubes are being greatly utilized in the chemotherapeutic field. 

### 6.2. Inorganic Nanoparticles

Inorganic nanoparticles (NPs) are those nano-carriers that are developed using various metals (e.g., copper, silver, iron), semiconductors, and carbon dots. They are being explored quite extensively recently for theranostics purposes in cancer research [173,174]. Nowadays, it has gained attention due to exclusive features such as good biocompatibility, hydrophilic nature, high stability, and wide surface conjugation chemistry over organic nanoparticles for better imaging and drug delivery [173,174]. Although, it has numerous advantages for cancer treatment a few inorganic NPs have been successfully transformed for clinical use due to photo-induced tissue damage, non-specific toxicity, and immunogenicity related challenges. Thus, complete research on safety, synthesis, pharmacokinetics, and bio distribution is required in the case of inorganic NPs [173,174]. Some brief details pertaining to different inorganic nanoparticles are mentioned below.

#### 6.2.1. Silica Nanoparticles

The silica nanoparticles are generally spherical. The natural silica itself is the crystalline base material in these nanoparticles. The nanoparticles can be modified into varied sizes and could be furthermore surface modified in order to target specifically. The nonporous form of these nanoparticles can be absorbent and abrasive in nature, on the other hand, mesoporous form is the one that is currently being used in drug delivery applications. The two types of silica base materials include Colloidal Silica and Colloidal Mesoporous Silica. Colloidal silicas are used to prepare nano drug carriers. These are synthesized specifically in basic environment (pH 7–10), whereas mesoporous silica is mainly silica precursors arranged into mesophases which are crystalline in nature. Further, these nanoparticles if surface modified using polymers, lipid bilayers are thus designed to load, retain, protect and release the drugs. Periodic mesoporous nanoparticles are synthesized from a sol-gel procedure [175]. Cheng and colleagues designed and applied the silica nanoparticles in a very unique manner against TNBC. The study included the designing of dendritic large pore mesoporous silica nanoparticles which were deposited with copper sulphide nanoparticles, and the immune adjuvant Resiquimod drug was also loaded in a controlled manner. These were further coated with the homogenous cancer cell membrane and thus conjugated with anti-PD1 peptide AUNP-12 by using a PEG linker, forming an acid-labile benzoic-imine bond. The final formulation was evaluated on metastatic TNBC in vitro as well as in vivo. It was observed that the designed nanoparticles were having high potential of targeting TNBC and also inducing photothermal ablation over primary TNBC tumors. This study thus enabled the pathway of using intelligent biomimetic nanoplatform in the treatment of metastatic TNBC [176]. In another study, the large pore mesoporous silica nanoparticles were deposited with a leukocyte/platelet hybrid and were also co-loaded with Doxorubicin. These silica-based nanoparticles were observed to showcase excellent TNBC-targeting ability as well as increased photothermal activity in vitro and in vivo. Thus, even this approach could be employed for the treatment of metastases [177]. The mesoporous silica nanoparticles could further be modified with RGD peptides and deposited with arsenic trioxide (ATO) in the treatment of MDA-MB-231 TNBC in vivo. The modified nanoparticles, mesoporous silica-RGD peptide-Arsenic trioxide, showcased a better therapeutic effect as compared to nonmodified silica nanoparticles, free arsenic trioxide, and arsenic trioxide combined with silica nanoparticles [178].

#### 6.2.2. Silver Nanoparticles

Although silver metal is being used for several years, silver nanoparticles are a recent development and are being considered for delivering therapeutics for the past few decades. Silver nanoparticles are being used in medicine as well as the agricultural field as antibacterial, antifungal, and antioxidant. In a broader sense, silver nanoparticles have varied applications as biomedical devices, nano drug carriers, imaging probes, etc. [179,180]. The silver nanoparticles were coated with albumin and were evaluated on MDA-MB-231, the TNBC cell line. The formulation ultimately demonstrated cell-based apoptosis, and reduction of gland tumor sizes in mice. Furthermore, it was observed from this study that LD50 of albumin coated Silver Nanoparticles was found to be 30 times more compared to that of normal white blood cells [181]. The systematically dosed silver nanoparticles were found to be effective even at non-toxic doses in repressing the growth of TNBC xenografts in mice [182]. It has been observed that the silver nanoparticles showed selective toxicity in TNBC cell lines even at particular concentrations which are non-lethal to non-cancerous cells. Silver nanoparticles hold the potential for inducing selective toxicity in and sensitizing the TNBC cells to ionizing radiation and also have the potential of PTT heat transducer. Thus, the results indicated that the developed silver nanoparticle plates were effective as selective cytotoxic multimodal therapy against TNBC cell lines and thus to decrease the viability of TNBC cell lines, the combination of silver nanoparticles, PTT as well as ionizing radiations as compared to individualized treatment [183]. 

#### 6.2.3. Gold Nanoparticles

Recent trends have seen an exploration of the therapeutic and cytotoxic potential of gold nanoparticles. Gold nanoparticles can be developed and synthesized in varied shapes and configurations such as Gold nanoshells, gold nanorods, gold nanocages for delivering cytotoxic drugs [110]. One of the studies conducted explored the mechanism behind the cytotoxicity against TNBC cells induced by differentially charged gold nanoparticles. The mechanism involved induction of oxidative stress, modification of WNT signaling pathway as well as epigenetic modifications, specifically histone H3 modifications at serine 10 and lysine 9/14 residues respectively. The presence of the type of charge also modified the duration for induction of cell death. The postive charges induced cell death abruptly whereas the negative charges slowly induced cell death in MDA-MB-231 cells [184]. Another way of harboring the cytotoxic properties of gold nanoparticles is the use of negatively charged gold NPs which bind to the cell-cell junction which is charged positively charged, thus inducing the formation of a leaky effect owing to the charge of the nanoparticles and thus enabling the access to cancer cells [110]. The gold nanoparticles also sensitize the TNBC cell line, MDA-MB-231 to cytotoxic drug 5-Fluorouracil via the mechanism of reducing the expression of thymidylatesynthetase [184]. In order to develop a treatment modality against TNBC, 21 nm gold nanoparticles were functionalized with Chlorine e6 and Epidermal Growth Factor (EGF). This bifunctionalization helped the specific targeting of TNBC cells mediated via PDT as well [185]. Thus, these gold nanoparticles can be explored furthermore as a nano vehicle as well as therapeutic agents against TNBC.

### 6.3. Organic-Inorganic Hybrid Nanocarriers

Organic/inorganic hybrid nanocarriers are established to offer the combinatorial benefits of both organic and inorganic nanoparticles. This includes good loading capacity, better release profile, biomimetic system, improved selectivity along with providing better therapeutic efficacy over the parent nanocarriers [186]. For example, surface fuctionalisation of Mesoporous Silica Nanoparticle (MSN) with polyethyleneimine (PEI) provides a contemporaneous benefit of improved cellular uptake of MSNs and also offers efficient nucleic acid delivery as PEI is a cationic polymer which imparts cationicity to the nanoparticle [187]. Manisha et al. developed a surface functionalized CD44 conjugated MSN for miRNA delivery using HA-PLGA for treating TNBC. It was reported that these surface coated MSN nanocarriers provide a better stability to the nanoparticle, have improved effective internalistion into the cellular environment, representing a stable and sustained release of microRNAs from the nanoparticle which further helps to kill the cancer cells [188]. In an another study, folic acid decorated metal organic framework nanocarriers were developed by Laha et al. for the treatment of TNBC [189]. This carrier provided greater uptake and sustained intracellular delivery of curcumion and also enhanced the the therapeutic effectiveness at the tumor site.

## 7. Stimuli Based Targeting Approaches in Nano Drug Delivery System for TNBC

**Figure 4:**
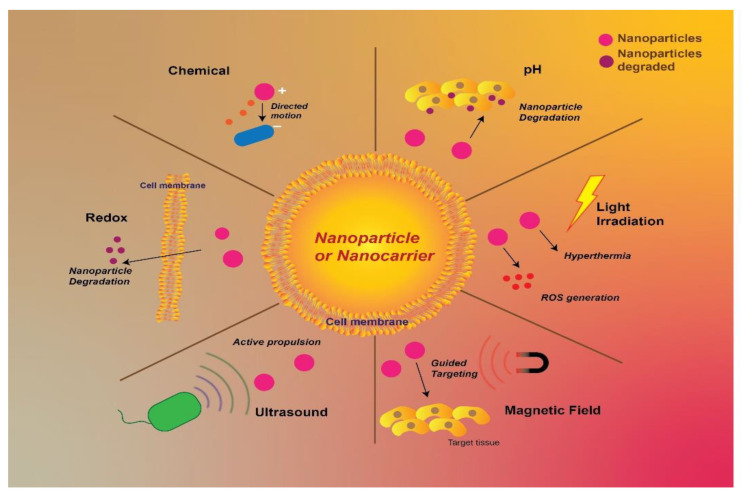
Different stimuli-responsive nanoparticles.

Stimuli-sensitive systems are the systems that are being explored for the benefit they provide, that is controlled and sustained drug delivery. The stimuli-sensitive systems release the drug based on the internal or external stimuli which can be encompassed under environmental stimuli. These stimuli-sensitive systems can be delivered through various routes, including parenteral, ocular, rectal, vaginal, and dermal as well as transdermal delivery systems [190]. The use of these stimuli-based release systems is currently being worked on for specific targeting and release against TNBC [59]. The stimulus for these stimuli-sensitive release systems can be of two forms, either internal (endogenous) or external (exogenous). The internal stimuli involve pH, GSH, and enzymes whereas the external stimuli involve light (laser beams), temperature, magnetic field, and ultrasound [191]. Among these, the utilization of high temperature is considered one of the best approaches as many medical researchers have found the beneficial uses of hyperthermia in the treatment of solid tumors [192]. A pictorial representation of different stimuli-based nanoparticles is shown in Figure 4. The employment of stimuli-sensitive nanocarriers enables the nanocarriers to actively participate in drug and gene delivery compared to the conventional use of nanocarriers just as vehicles. In order to get the desired stimuli-sensitive nanocarriers, the composition of the nanocarrier assemblies could be modified accordingly. Another advantage of using the stimuli-sensitive nanocarriers is that the stimuli which can be harnessed for the release of the drug are specific to that tumor or disease pathology itself. This allows the modified stimuli-sensitive nanocarrier to release the drug over specific pathological triggers only [193]. Furthermore, if the TME is explored and understood in a detailed manner, it will enable the development and clinical use of the stimuli-sensitive systems, responding to abnormal conditions of the tumor tissues.

Overexposure to the specific stimulus, would convert these stimuli-responsive systems into controlled drug-releasing systems or would also enhance the uptake or penetration of the drug in the tumor microenvironment. The engineering of the stimuli-sensitive drug delivery systems, mainly relies on the specific biochemical contents of the affected areas and thus leads to specific temporal and spatial drug delivery [194,195,196]. Some recently reported stimuli-based nanoformulations and their outcome are displayed in Table 1.

### 7.1. pH Responsive Delivery System

pH responsive drug delivery system works to protect the drug in blood at pH 7.3–7.4 whereas this system releases the drug or expresses other functions as enhancement of cell penetration potential in the TME with pH 6.8–7.2. This pH-sensitive modified system can also release the loaded drug in response to changes in pH in the cytoplasm (pH greater than 7), endosomes (pH 5–6), and lysosomes (pH 4.5–5.5). If a higher concentration of the drug is localized in the tumor cell cytoplasm, this would enable the increased cytotoxic property of the drug and thus overcome the Pgp efflux capacity and ultimately have a cytotoxic effect on tumor cells [210,211]. Hence, the pH-responsive drug delivery systems exploit the difference in the pH of the cancer tissues and that of the normal tissues, and the acidic pH in endosomal and lysosomal regions for the release of chemotherapeutic drugs. 

The strategy used for these pH-responsive drug delivery systems is that upon being triggered by an altered pH (stimuli), they result in modifications in the polymer of nanocarrier in terms of its physical properties like shape, size, or its nature of hydrophobicity [211]. There are two approaches that are being explored in order to achieve pH-responsive release in the TME. One of the approaches is the use of pH labile chemical bonds while conjugation of the drugs with polymers for example use of pH-sensitive polymer-drug conjugates. Examples of such linkages are hydrazone linkage, cis acotinyl linkage as well as acetal linkages. These acid-labile linkages are stable at the physiological pH, neutral pH, or alkaline pH but would break in the acidic pH (of the TME) [193,194,212]. 

In one such attempt of the application of hydrazone linkages, Patil et al. designed Doxorubicin conjugated PEG nanoparticles via pH-sensitive hydrazone linkages to a nanoconjugate platform of Poly (β–L-malic acid). It was observed that the pH sensitive conjugates were stable in physiological pH whereas released the loaded drug and inhibits in vitro cancer cell growth of MDA-MB-468 and MDA-MB-231 cell lines [197]. Furthermore, another application is the use of self-healable, injectables, which are pH responsive in nature and are the hybrid hydrogels formed via the formation of hydrazone linkages between hydrazide functionalized gelatin (Gel-ADH) and Aldehyde functionalized PEG polymers. The researchers investigated this modified pH-responsive hydrogel against cell lines MDA-MB-231 and observed the potential use of the modified hydrogel in the same [198]. 

Another approach is the use of copolymers involving titratable groups such as carboxylic acids, amines, which have the potential to get protonated and thus regulate the micelle formation based on the alteration of pH. The change in pH induces micelle formation and thus induces dissociation or alteration in the internal structure of the formulation. As the pH varies, this causes protonation of the pH-sensitive polymers which at normal physiological pH forms a hydrophobic core. As the pH lowers, with lower pKa protonatable groups get charged which result in destabilization and separation of the polymeric chain from the micellar chain. Examples of such protonatable polymers are poly(histidine), poly(acrylicacid), polysulfonamides [211]. Various researchers explore the use of poly (histidine) in the formulation of pH-sensitive systems against the tumor cells. In one such attempt, Hwang et al. explored the use of copolymer dextran-b-poly (histidine) in the formulation of Doxorubicin loaded nanoparticles via the nanoprecipitation method. From the study, it was observed that the nanoparticle releases the drug in the acidic tumor pH, in a pH-dependent controlled release manner [199]. Furthermore, Ko and colleagues designed Camptothecin and Tetramethylrhodamine (TRiTC) in MPEG Poly micelles. This formulation showcased micelle formation and destabilization at pH 6.8, with a pH-dependent drug release. These Camptothecin loaded micelles were evaluated for their cytotoxicity on MDA-MB-231 cell lines. In vivo, the tetramethylrhodamine loaded MPEG micelles were observed to be 11 times more accumulative compared to Tetramethylrhodamine conjugated Camptothecin without pH-sensitive micelles [200]. 

### 7.2. Temperature-Sensitive Delivery System

One of the characteristics of the tumor pathological tissues is local hyperthermia. This difference in temperature of normal healthy tissues and the tumor tissues can be harnessed and thus exploited for triggering the release from temperature-sensitive drug delivery system. Furthermore, so as to regulate the temperature for specific locations for the release of the drug, external heat sources could be used. These external heat sources include dielectric heating using microwave induction, ultrasound application, heating using an electrode with higher frequencies, heating using fiberoptics, laser photocoagulation as well as water bath heating [213,214]. The heating of the tumor location results in an increase in the size of the endothelial pore as well as there is an increase in the blood flow to that site, leading to enhanced extravasation of the nanocarriers [196]. The usage of certain lipids with a specific gel-to-liquid transition temperature is one of the approaches used for temperature-sensitive drug delivery. At that specific transition temperature, the liposomal membrane destabilizes, and the drug releases at temperatures of 40–42 °C in clinical hyperthermia protocols. One such good example of the liposomal composition is use of DPPC/MSPC/DSPE-PEG2000 in the ratio of 90:10:4 mole ratio [215,216]. Another most studied lipid for this temperature-sensitive drug delivery systems is dipalmitoylphosphatidylcholine (DPPC), having the transition temperature gel to liquid form of 41 °C [217,218]. In an attempt to use this temperature-sensitive drug delivery system, a solution was developed over the nanoplatform of thermo-sensitive poly (N-vinylcaprolactam)-chitosan nanoparticles. This was altered by the addition of cell-penetrating peptide and loading of the cytotoxic drug Doxorubicin. At the increased temperature of the TME, the base copolymer would lead to phase change, resulting in drug release and thus localization of the cytotoxic drug in the tumor tissues. This modified formulation resulted in decreased off-target cytotoxicity compared to the free drug form of Doxorubicin. It was observed in vivo that this formulation was effective in significantly reducing the tumor volume with decreased systemic side effects [201,219]. In a similar type another study, YC. Ou et al. developed a PTT based gold nanoparticle against the TNBC cells. In this work, a multi-branched gold antenna facilitates elevated temperature via the conversion of NIR light to heat and thus, delivers doxorubicin effectively to the cancer tissue. The modified combination therapy designed in this study, showcased enhanced cytotoxicity in TNBC cell lines compared to the free drug Doxorubicin, with decreased off-target cytotoxicity [220]. 

### 7.3. ROS Responsive

ROS responsive nanocarriers are those nanocarriers, which alter their properties based on the presence of ROS. Exposure to in vivo ROS leads to change (physical or chemical) in the ROS-responsive nanocarriers. These ROS-responsive nanocarriers have certain specific advantages compared to conventional nanocarriers. Such ROS-responsive agents can have varied applications including, imaging agents, and site-specific delivery of cytotoxic drugs. These ROS-responsive agents could also modify the tissue microenvironment [221,222,223]. So as to achieve increased drug localization in tumor tissues, Xi et al. developed redox-responsive diselenide containing dipeptide and further co-precipitated them with amphiphilic co-polymers, which has the potential to selectively target TNBC. This modified formulation exhibited increased localization of the drug in TNBC cells with decreased systemic toxicity in both in vitro and in vivo experiments [202]. In another study, the authors investigated a unique form of nanoparticles, which activate over the Fenton reaction. These nanoparticles contain polymers that are ROS responsive and thus also activate the cascade biological reaction of ROS in tumor cells, The significant increase in the elevated level of ROS resulted in an evident antitumor effect in vitro via expression of cytochrome c, caspase-9, and caspase-3 as well as inhibition of Matrix Metallo Protein–9, thus promoting apoptosis and inhibiting metastasis [203]. 

### 7.4. Enzyme-Responsive Drug Delivery System

The enzyme-responsive drug delivery system, in presence of an enzyme, undergoes various structural/physical changes via biocatalytic actions of the specific enzymes. The TME has the presence of certain specific upregulated enzymes, which enhances the potential use of enzymes responsive drug delivery system. The enzyme-responsive drug delivery systems, has unique properties including, sensitivity, selectivity, catalytic efficacy, and biorecognition [224]. The polymeric content of the formulation degrades and releases the drug upon the action of a specific enzyme. 

**Figure 5:**
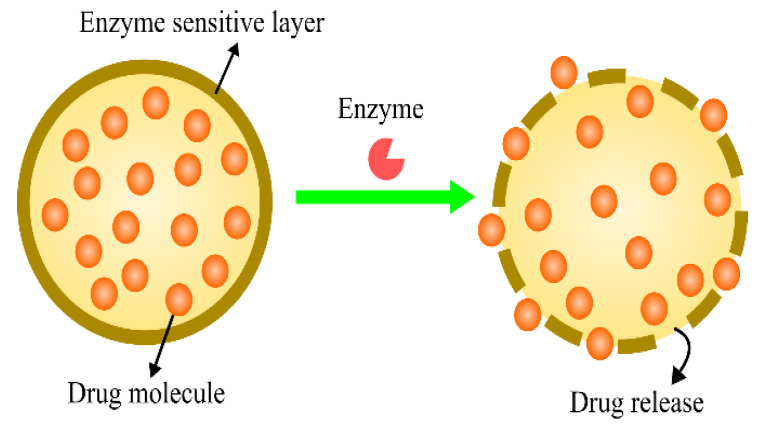
Enzyme responsive drug delivery system.

Proteases are the enzymes, which are gaining attention in order to develop the enzyme-responsive drug delivery system. This is because the proteases are often overexpressed in terminal diseases like cancer [225]. In one such attempt of targeting tumor cells, Radhakrishnan et al. designed dual enzyme-responsive nanocapsules loaded with anticancer drugs for their intracellular delivery. The enzyme trypsin or hyaluronidase, causes the capsule wall of polymer to degrade, leading to intracellular delivery of anticancer drug in the tumor tissue [204]. A representative diagram for enzyme responsive drug delivery system has shown in Figure 5. Moreover, in another study that was conducted by Liu et. al., they fabricated an enzyme responsive liposome for co-delivery of tariquidar and doxurubicin to eradicate TNBC using mmp-2 enzyme. This targeted nanocarrier provided potential therapeutics to control tumor growth in drug-resistant TNBC via EGFR targeting and also exhibited controlled release, and prevent transporter efflux which helps the drug to be bioavailable at the tumor site. This study suggested that enzyme stimuli based targeted nanoplatform has the potential to treat drug-resistant TNBC and also enhanced the penetration of drug into the TME [226].

### 7.5. Magnetic Responsive Drug Delivery Systems

The magnetic rays have the potential to penetrate into the body and thus are used for body imaging in MRI [227]. These magnetic-responsive drug delivery systems are used for the controlled release of drug targeting the tumor sites. The peculiar characteristics of these systems include biocompatibility, biodegradability, easy synthesis as well as modification [228]. These are potential drug delivery systems as they have a smaller size and thus could easily target the tumor cells. In general, the mechanism involves the generation of heat by the magnetic responsive drug delivery systems, using alternating magnetic frequency. The magnetic drug delivery systems, are being utilized in the therapeutic area, on the basis of two different mechanisms, involving magnetic field-guided drug targeting and magnetic field-induced hyperthermia [206,229]. The hyperthermia-based mechanism has been widely applied to deliver drugs to cancer cells. Furthermore, the resulting hyperthermia via magnetic response also leads to inhibition of the tumor cells as well as provides an opportunity for imaging scans. In an attempt to explore the application of magnetic-responsive drug delivery systems [230]. Wang et al. developed an implantable magnetic chitosan hydrogel which was loaded with the drug Rifampicin, a hydrophobic drug, and Adriamycin, a hydrophilic. The external stimuli applied here was a very low frequency alternating magnetic field which lead to a pulsatile drug release from the delivery system with no involvement of hyperthermia associated with a magnetic field. The authors were successful in developing a magnetic responsive controlled release drug delivery system [205]. Thirunavukkarasu et al. developed superparamagnetic iron oxide nanoparticles, which were loaded with Doxorubicin in a PLGA matrix. This PLGA responds to the application of a magnetic field, leading to the subsequent release of Doxorubicin at specific tissue. The iron oxide nanoparticles undergo a change, leading to a change in the temperature of the solution, which further causes the transition in PLGA, ultimately leading to the release of the drug Doxorubicin [206].

### 7.6. Electrical Responsive Drug Delivery System

Electro-responsive drug delivery systems act as a delivery system with an external electric field, the drug from the nanoparticle leads to release after the application of a weak electric field. There are various mechanisms, behind the controlled drug release via electrical stimulation, consisting of an oxidation-reduction reaction, degradation of carrier structure, and stimulation of thermo-responsive carriers [231,232,233]. Xie et al. developed an electroresponsive polydopamine-polypyrrole microcapsules for dexamethasone delivery. The electroresponsive mechanism behind the release was the redox mechanism of polypyrrole. Furthermore, the nanoformulation also was observed to showcase, increased bioavailability, higher drug loading as well as increased cell adherence ability [207]. Neumann et al. used another approach via the application of electrical stimuli. The authors utilized the alterations in the pH change as a result of external electrical stimuli for regulating the release of the drug. The pH-sensitive drug-polymer used was poly(methyl methacrylate-co-methacrylic acid). In addition to this, the authors also observed that pH alterations were reversed after the removal of the external electrical stimuli thus leading to the stopping of the drug release [234]. 

### 7.7. Ultrasound-Responsive Drug Delivery Systems

The ultrasound-responsive drug delivery systems have unique advantageous properties, including non-invasiveness, safety, and tissue penetration [235]. The three forces, thermal, mechanical as well as radiation forces are the forces behind the release from ultrasound responsive drug delivery system [236]. In a similar attempt, to use these unique ultrasound responsive drug delivery systems, Xin et al. developed such nanoparticles using PLGA, for inducing ultrasound-responsive vibrations, leading to disruption of the liposomal membrane [208].

Paris et al. developed mesoporous silica nanoparticles which were PEGylated via thermosensitive linker 4,4′-azobis(4-cyanovaleric acid). This thermosensitive linker cleaved upon application of external stimuli of ultrasound waves, leading to drug release protected by PEGylation. This also gave a positive charge to the silica nanoparticles, which in turn increased the cellular uptake [209]. In another study, the researchers developed Ultrasound responsive liposomes using PLGA, which were loaded with the drug Mitoxantrone, which increased the drug release approximately up to 90% compared to the non-ultrasound responsive release of approximately 50%. Furthermore, this ultrasound-responsive liposomal nano-formulation showcased increased blood half-life time, making it a potential drug delivery system [208,237]. 

### 7.8. Hypoxia Responsive Drug Delivery Systems

Hypoxia is one of the crucial parameters driving the cellular mechanisms in TME. Primarily, it is involved in tumor angiogenesis, invasion, metastasis and immunosuppression [238]. Due to this evidences, researchers have an increased interest towards developing a target-based nanomaterial for hypoxic TME. Liu et al. developed a hypoxia responsive self-assembled micelle of doxorubicin and ICG using NIDH (nitroimidazole hexylamine) block copolymers. This nanocarrier is capable of detecting hypoxia in the TME to trigger the cytotoxicity and immunogenic responses towards the effective treatment of 4T1 breast cancer. This work suggested that this targeted long-acting nanocarrier was utilized to diagnose the triple negative 4T1 breast tumors via photoacoustic imaging technique, and successfully killing tumors without recurrence [239]. In another research work, Zhang et al. developed a hypoxia responsive drug-drug conjugated nanoparticle using chemical linker Azobenzene for three drugs (combretastatin A-4, irinotecan and cyclopamine) to treat MCF-7 cancer. This carrier could effectively trigger the apoptosis and prevent the proliferation and differentiation cancer. Also it could improve the the cellular uptake and the permeability of the drug [240]. These work demonstrated that this nanocarrier provided a promising therapeutic strategy for tumor management.

## 8. Regulatory Considerations of Nanoformulations for TNBC

Nowadays, the nanomaterial application in drug delivery has amplified, with many different advanced nano formulation approaches being exploited within the clinical translation. These nanomaterials have numerous unique characteristics which help them in clinical applications. One of its unique properties is narrow range particle size i.e., 10–200 nm which permits the nanoparticle for systemic circulation over a long period of time and also helps to avoid clearance via renal and complement systems [241,242]. This feature of nanoparticles provides a momentous approach to the drug for cancer treatment as it was shown high EPR effect and greater penetration of the drug into the cancer microenvironment. Furthermore, Surface modification of these nanoparticles with various biomarkers including protein, aptamer, folic acid, RGD, etc. delivers site-specific targeting and selectivity characteristics toward the treatment of cancer. One more important characteristic of nanoparticles is electronic and optical characteristics, predominantly in metallic nanoparticles [243]. These characteristics are permitting the nanoparticles to be exploited for numerous applications including bio-imaging, effective targeting, and drug delivery. However, pertaining to nanomaterials, the scientific community has very high expectations regarding disease treatments, still, a lot of obstacles are being observed in the path of development of nano-therapeutics as a standard regulatory guideline for nanomedicine is not available. Despite the absence of regulatory guidelines, there are several nanoparticles already established in the market. Mostly, these are used for cancer treatment; examples of these nanoparticles include Doxil^®^, AmBisome^®^, Abraxane^®^, etc. [21,244]. Additionally, there are some nanomaterials have been under clinical trials for TNBC (Table 2). Although these studies are showing potential effectiveness against TNBC, still their production, scale up and safety is other concerns. Lack of standardization and scientific expertise makes it difficult for the regulatory agency to monitor the safety and effectiveness of these nanomaterials.

Moreover, most of the nanomedicines are working by interacting with genetic materials or biochemical which is participated in normal genome function and cellular activity. All of these nanoparticles can produce severe toxicity to the body and also can cause cancer via various abnormal pathways i.e., oxidative and nitrosative stress, lipid peroxidation, oxidative DNA damage etc. Therefore, regulatory agencies and the research community is need to work on the development a standard guideline [245]. Currently, USFDA, Health and Consumer Protection Directorate of the European Commission and US-EPA have engaged so as to deal with adverse effect by nano-therapeutics. But there are numerous challenges in the way of the progression of guidelines. One of the major challenges in the regulation of nanomedicine is regulatory bodies i.e., USFDA practice safety data sheet for bulk materials that do not show a similar pharmacokinetic and efficacy profile as in nanomedicine. it can create a severe issue on safety and efficacy of nano-therapeutics. Other issues related to manufacturing, scale-up, and stability, as the nanomedicine is having complex structure and characteristics, it is highly tedious to prepare a consistent batch. Thus, critical Quality Attributes (CQA) is required to monitor the screening of raw material, manufacturing process, scale up and stability that can support understanding nanomedicine. During the clinical translation, the drug mechanism and preclinical safety profile of nanomedicine is needed before approval of clinical trials. Otherwise, it may be compromised the safety and effectiveness of the product. All of the above-discussed challenges are restricting the nanomedicine for their future development.it can directly deter product safety, and quality of products, and could be leading to unproductive control of nanoparticles due to the absence of product-specific safety protocol [21,244,246]. Therefore, to establish the efficient benefits accessed by nano-therapeutics against cancers or other life-threatening diseases, many critical concerns on the regulatory protocol is need to be addressed by the regulatory bodies.

## 9. Conclusions and Future Perspectives

Triple-negative breast cancer is a heterogeneous metastatic form of breast cancer with unique biological features. Its aggressiveness, poor prognosis, and high rate of recurrence make its management highly challenging. Conventional approaches such as use of cytotoxic chemotherapy (taxanes, anthracyclines, or platinum agents), surgery, and radiotherapy were widely being evaluated by the researchers for their effectiveness in triple-negative breast cancer. However, these therapies involve certain limitations, such as multidrug resistance, poor bioavailability, unwanted side effects, and poor prognosis. Scientists also developed targeted therapies, including EGFR inhibitors, VEGF inhibitors, mTOR inhibitors, and PARP inhibitors. Despite being specific in their action, resistance offered by cancer cells to these inhibitors has hindered their use for TNBC. Nanotherapeutics have surmounted the difficulties of traditional and targeted therapies. They selectively target tumor cells by enhanced permeation and retention effect or by active targeting. Encapsulation of anti-cancer drugs in these nanocarriers protects them from the external environment and enhances their biological half-life, decreasing the dose required for the desired effect. Researchers have developed several nanoparticles for treating as well as monitoring cancer, making them promising delivery systems. Further, various ligands can be attached to the nanocarriers to target them to the desired site. Various organic and inorganic nanoparticles have been developed for TNBC. siRNA and miRNA have also gained the attention of researchers as a therapeutic option for cancer. Delivery of such organic particles is the current need of scientists as TNBC cells lack biomarkers and unifying molecular features. Characteristics of the TME have led to the progress of immunotherapy in TNBC. In another perspective of employing immunotherapy, nanocarriers are employed to carry agents targeting the immune components involved in TNBC progression. However, they have to cross the biological barriers that constitute the natural defense system of our body. Further, their difficult scale-up, efflux by the transporter, and regulatory approval limit their use for successfully treating cancers. There is a need for universities, governments, and pharmaceutical industries to join hands and work on regulatory guidelines for nanotherapeutics. 

In the future, precision nanomedicine, which takes into account genetic differences as well as physiological and pathological variabilities amongst patients will be the focus of scientists. Successfully developing novel targeted nanomaterial can be utilized in both treatment and diagnosis of various diseases which will provide a promising future in cancer research. These nanomaterials deliver lot of advantages such as possibility to load/encapsulate drug which can offers stability to the drug from the biological system. As a result, it could enhance biological half-life of anti-cancer drug. Further, these can deliver better targetability to tumor tissue, greater transfection towards cancer cell, as it could facilitate its conjugation with various biomarkers (Protein, ligand, nucleic acid, stimuli-responsive chemical etc.). In contrast, these nanomaterials have shown lot of challenges such as immunogenicity, lack of understanding of nanomaterial on molecular and cellular interaction and lack of clinical translation due to limited availability of TNBC (cell or animal) model. Thus, there is requirement of innovative therapeutic approaches in the future. At the same time, attention has to be given towards developing toxicity-free nanoparticles with the capability of targeting desired tissue and exhibit desired pharmacokinetics selectively. Understanding the complex heterogeneity of TNBC and the identification of new biomarkers that can be targeted will effectively treat TNBC. Further progress in the field of nanotechnology may guarantee success against this life-threatening cancer.

## Figures and Tables

**Table 1:** Stimuli-based targeting approaches in nano drug delivery system for TNBC.

S.No.	Type of Stimulus	Polymer/Nanoplatform	Drug	Significant Outcome	References
1.	pH (internal)	PEG and Poly (β–L–malic acid)	Doxorubicin	Inhibits in vitro cell growth in MDA-MB-468 & MDA-MB-231	[197]
2.	pH (internal)	Aldehyde functionalized PEG polymers	Doxorubicin	Hydrogel inhibited invitro cell growth in MDA-MB-231	[198]
3.	pH (internal)	Dextran-b-poly(histidine)	Doxorubicin	Viability of tumor cells decreased upon pH sensitive controlled release of drug	[199]
4.	pH (internal)	MPEG-poly-(beta-amino ester)	Camptothecin and Tetramethylrhodamine	Invitro significantly inhibited the growth in 5.MDA-M6.B-231 and 7 in vivo 1 time more accumulative compared to non pH sensitive micelles	[200]
5.	Temperature (internal)	poly(*N*-vinylcaprolactam) (PNVCL)-chitosan	Doxorubicin	Decreased off target activity compared to free drug, Invivo reduced tumor volume with decreased systemic side effects	[201]
6.	Reactive Oxygen Species (internal)	Amphiphilic Synthetic Polymers: mPEG and *L*-phenylalanine *N*-carboxyanhydride	Paclitaxel	Increased localization, decreased systemic toxicity in both in vitro and in vivo	[202]
7.	Reactive Oxygen Species (internal)	PLGA	Doxorubicin	Significant increment in ROS levels, increased cytotoxic effect in vitro via expression of cytochrome c, caspase-9, caspase-3, MMP-9	[203]
8.	Enzyme (internal)	Chondroitin sulfate	Doxorubicin hydrochloride	Significant increase in cellular uptake as well improved release profile of the nanoparticles loaded with the drug	[204]
9.	Magnetic field (external)	Chitosan	Rifampicin and Adriamycin	Pulsatile, controlled release of the drug	[205]
10.	Magnetic field (external)	PLGA	Doxorubicin	Controlled release of the cytotoxic drug, Doxorubicin	[206]
11.	Electrical (external)	Polydopamine-Polypyrrole	Doxorubicin	Increased bioavailability, higher drug loading, increased cell adherence	[207]
12.	Ultrasound (external)	PLGA	Mitoxantrone	Increased drug release up to 90%, enhanced controlled release, enhanced blood half-life time	[208]
13.	Ultrasound and thermal (external)	PEG and 4,4-azobis(4-cyanovaleric acid)	Topotecan	Increased circulation time and cellular uptake	[209]

**Table 2:** Key Clinical trials and their outcomes which aimed to explore the nanotechnology-based approaches against TNBC.

Intervention	Nanocarrier	Outcome	Status & Primary Completion Date	Company	NCT & Reference
Docataxel	Nanosomal Liquid Suspension	Proportion of the patients with Objective Response Rate (i.e., CR + PR) as the Best Overall Response Rate (i.e., CR + PR) in the test arm (NDLS) compared to reference arm (Taxotere) Progression free survival (PFS) To evaluate the overall survival (OS) of the patients Incidence of adverse events as assessed by clinical examination, and/or laboratory parameters	Phase III, 31 March 2022	Jina Pharmaceuticals, Intas Pharmaceuticals, Lambda Therapeutic Research Ltd.	NCT03671044
HLX10	In combination with Chemotherapy (as Neoadjuvant Therapy)	Tumor assessment	Phase III, 7 September 2022	Shanghai Henlius Biotech	NCT04301739
Atezolizumab + Nab-Paclitaxel	Nanoparticle albumin bound paclitaxel	Percentage of Participants with treatment-emergent Grade ≥ 3 AEs Percentage of Participants with treatment-emergent Grade ≥ 2 imAEs Percentage of Participants with all treatment-emergent AEs	Phase III, 30 June 2022	Hoffmann-La Roche	NCT04177108
Nab- Paclitaxel & Bevacizumab	Nanoparticle albumin bound paclitaxel	Progression free survival. Response rate. Overall survival. Safety and toxicity. Exploratory biomarkers will be assessed as potential predictors of response to treatment including: expression of epidermal growth factor receptor (EGFR) and secreted protein acidic and rich in cysteine (SPARC) in the primary tumor and changes in levels of circulating tumor cells (CTCs) and circulating endothelial cells (CECs).	Phase II, 5 July 2017	Sponsor: University of Washington Collaborator: National Cancer Institute (NCI)	NCT00733408
CORT125134 in Combination with Nab-paclitaxel	Nanoparticle albumin bound paclitaxel	Maximum Tolerated Dose of CORT125134 in Combination with nab-paclitaxel, Number of Treatment-Related Adverse Events as Assessed by CTCAE version 4.0 for Patients with Solid Tumors Treated with CORT125134 in combination with nab-paclitaxel	Phase I/II, May 2020	Corcept Therapeutics	NCT02762981

## Data Availability

Not applicable.

## References

[1] Anders C., Carey L.A. (2008). Understanding and treating triple-negative breast cancer. Oncology.

[2] Shekar N., Mallya P., Gowda D.V., Jain V. (2020). Triple-negative breast cancer: Challenges and treatment options. Int. J. Res. Pharm. Sci..

[3] Dietze E.C., Carolina N., Carolina N., Seewaldt V.L. (2017). Triple-negative breast cancer in African-American women: Disparities versus biology. Nat. Rev. Cancer.

[4] Lee E., McKean-Cowdin R., Ma H., Spicer D.V., Van Den Berg D., Bernstein L., Ursin G. (2011). Characteristics of triple-negative breast cancer in patients with a BRCA1 mutation: Results from a population-based study of young women. J. Clin. Oncol..

[5] Neven P., Brouckaert, Wildiers, Floris (2012). Update on triple-negative breast cancer: Prognosis and management strategies. Int. J. Womens Health.

[6] Yin L., Duan J.J., Bian X.W., Yu S.C. (2020). Triple-negative breast cancer molecular subtyping and treatment progress. Breast Cancer Res..

[7] Burstein M.D., Tsimelzon A., Poage G.M., Covington K.R., Contreras A., Fuqua S.A.W., Savage M.I., Osborne C.K., Hilsenbeck S.G., Chang J.C. (2015). Comprehensive genomic analysis identifies novel subtypes and targets of triple-negative breast cancer. Clin. Cancer Res..

[8] Mendes T.F.S., Kluskens L.D., Rodrigues L.R. (2015). Triple Negative Breast Cancer: Nanosolutions for a Big Challenge. Adv. Sci..

[9] Lehmann B.D., Jovanović B., Chen X., Estrada M.V., Johnson K.N., Shyr Y., Moses H.L., Sanders M.E., Pietenpol J.A. (2016). Refinement of triple-negative breast cancer molecular subtypes: Implications for neoadjuvant chemotherapy selection. PLoS ONE.

[10] Singhvi G., Rapalli V.K., Nagpal S., Dubey S.K., Saha R.N., Daima H.K., Pn N., Ranjan S., Dasgupta N., Lichtfouse E. (2020). Nanocarriers as Potential Targeted Drug Delivery for Cancer Therapy. Nanoscience in Medicine.

[11] Penault-Llorca F., Viale G. (2012). Pathological and molecular diagnosis of triple-negative breast cancer: A clinical perspective. Ann. Oncol..

[12] Bosch A., Eroles P., Zaragoza R., Viña J.R., Lluch A. (2010). Triple-negative breast cancer: Molecular features, pathogenesis, treatment and current lines of research. Cancer Treat. Rev..

[13] Roy R., Chun J., Powell S.N. (2011). BRCA1 and BRCA2: Different roles in a common pathway of genome protection. Nat. Rev. Cancer.

[14] Khan M.A., Jain V.K., Rizwanullah M., Ahmad J., Jain K. (2019). PI3K/AKT/mTOR pathway inhibitors in triple-negative breast cancer: A review on drug discovery and future challenges. Drug Discov. Today.

[15] Medina M.A., Oza G., Sharma A., Arriaga L.G., Hernández J.M.H., Rotello V.M., Ramirez J.T. (2020). Triple-negative breast cancer: A review of conventional and advanced therapeutic strategies. Int. J. Environ. Res. Public Health.

[16] Engebraaten O., Vollan H.K.M., Børresen-Dale A.L. (2013). Triple-negative breast cancer and the need for new therapeutic targets. Am. J. Pathol..

[17] Podo F., Buydens L.M.C., Degani H., Hilhorst R., Klipp E., Gribbestad I.S., Van Huffel S., van Laarhoven H.W.M., Luts J., Monleon D. (2010). Triple-negative breast cancer: Present challenges and new perspectives. Mol. Oncol..

[18] Borri F., Granaglia A. (2021). Pathology of triple negative breast cancer. Semin. Cancer Biol..

[19] Reis-Filho J.S., Tutt A.N.J. (2008). Triple negative tumours: A critical review. Histopathology.

[20] Elias A.D. (2010). Triple-negative breast cancer: A short review. Am. J. Clin. Oncol. Cancer Clin. Trials.

[21] Hejmady S., Pradhan R., Alexander A., Agrawal M., Singhvi G., Gorain B., Tiwari S., Kesharwani P., Dubey S.K. (2020). Recent advances in targeted nanomedicine as promising antitumor therapeutics. Drug Discov. Today.

[22] Wahba H.A., El-hadaad H.A. (2015). Current approaches in treatment of triple-negative breast cancer Treatment modalities of TNBC. Cancer Biol. Med..

[23] Nedeljkovi M. (2019). Mechanisms of Chemotherapy Resistance in Triple-Negative Breast Cancer—How We Can Rise to the Challenge. Cell.

[24] Andreopoulou E., Sparano J.A. (2013). Chemotherapy in Patients with Anthracycline- and Taxane-Pretreated Metastatic Breast Cancer: An Overview. Curr. Breast Cancer Rep..

[25] Anampa J., Makower D., Sparano J.A. (2015). Progress in adjuvant chemotherapy for breast cancer: An overview. BMC Med..

[26] Burotto M., Wilkerson J., Stein W.D., Bates S.E., Fojo T. (2019). Adjuvant and neoadjuvant cancer therapies: A historical review and a rational approach to understand outcomes. Semin. Oncol..

[27] Perez E.A., Moreno-Aspitia A., Thompson E.A., Andorfer C.A. (2010). Adjuvant therapy of triple negative breast cancer. Breast Cancer Res. Treat..

[28] Montemurro F., Nuzzolese I., Ponzone R. (2020). Neoadjuvant Or Adjuv. Chemother. Early Breast Cancer?. Expert Opin. Pharmacother..

[29] Akashi-Tanaka S., Watanabe C., Takamaru T., Kuwayama T., Ikeda M., Ohyama H., Mori M., Yoshida R., Hashimoto R., Terumasa S. (2015). BRCAness predicts resistance to taxane-containing regimens in triple negative breast cancer during neoadjuvant chemotherapy. Clin. Breast Cancer.

[30] Inaji H., Komoike Y., Motomura K., Kasugai T., Koyama H. (2002). The role of neoadjuvant chemotherapy for breast cancer treatment. Gan. Kagaku Ryoho..

[31] Gerber B., Loibl S., Eidtmann H., Rezai M., Fasching P.A., Tesch H., Eggemann H., Schrader I., Kittel K., Hanusch C. (2013). Neoadjuvant bevacizumab and anthracycline-taxane-based chemotherapy in 678 triple-negative primary breast cancers; results from the geparquinto study (GBG 44). Ann. Oncol..

[32] Wu J., Li S., Jia W., Su F. (2011). Response and prognosis of taxanes and anthracyclines neoadjuvant chemotherapy in patients with triple-negative breast cancer. J. Cancer Res. Clin. Oncol..

[33] Fisher C.S., Ma C.X., Gillanders W.E., Aft R.L., Eberlein T.J., Gao F., Margenthaler J.A. (2012). Neoadjuvant chemotherapy is associated with improved survival compared with adjuvant chemotherapy in patients with triple-negative breast cancer only after complete pathologic response. Ann. Surg. Oncol..

[34] Kim K., Park H.J., Shin K.H., Kim J.H., Choi D.H., Park W., Ahn S.D., Kim S.S., Kim D.Y., Kim T.H. (2018). Breast conservation therapy versus mastectomy in patients with T1-2N1 triple-negative breast cancer: Pooled analysis of KROG 14-18 and 14-23. Cancer Res. Treat..

[35] Guo L., Xie G., Wang R., Yang L., Sun L., Xu M., Yang W., Chung M.C. (2021). Local treatment for triple-negative breast cancer patients undergoing chemotherapy: Breast-conserving surgery or total mastectomy?. BMC Cancer.

[36] Yao Y., Chu Y., Xu B., Hu Q., Song Q. (2019). Radiotherapy after Surgery Has Significant Survival Benefits for Patients with Triple-Negative Breast Cancer. Cancer Med..

[37] Baskar R., Lee K.A., Yeo R., Yeoh K.W. (2012). Cancer and radiation therapy: Current advances and future directions. Int. J. Med. Sci..

[38] Abdulkarim B.S., Cuartero J., Hanson J., Deschênes J., Lesniak D., Sabri S. (2011). Increased risk of locoregional recurrence for women with T1-2N0 triple-negative breast cancer treated with modified radical mastectomy without adjuvant radiation therapy compared with breast-conserving therapy. J. Clin. Oncol..

[39] Bellon J.R., Burstein H.J., Frank E.S., Mittendorf E.A., King T.A. (2020). Multidisciplinary Considerations in the Treatment of Triplegative Breast Cancer. Can. CA. Cancer J. Clin..

[40] He M.Y., Rancoule C., Rehailia-Blanchard A., Espenel S., Trone J.C., Bernichon E., Guillaume E., Vallard A., Magné N. (2018). Radiotherapy in triple-negative breast cancer: Current situation and upcoming strategies. Crit. Rev. Oncol. Hematol..

[41] Rosenblum D., Joshi N., Tao W., Karp J.M., Peer D. (2018). Progress and challenges towards targeted delivery of cancer therapeutics. Nat. Commun..

[42] Barua S., Mitragotri S. (2014). Challenges associated with Penetration of Nanoparticles across Cell and Tissue Barriers: A Review of Current Status and Future Prospects. Nano. Today.

[43] Lin J., Miao L., Zhong G., Lin C.-H., Dargazangy R., Alexander-Katz A. (2020). Understanding the synergistic effect of physicochemical properties of nanoparticles and their cellular entry pathways. Commun. Biol..

[44] Zhao Z., Ukidve A., Krishnan V., Mitragotri S. (2019). Effect of physicochemical and surface properties on in vivo fate of drug nanocarriers. Adv. Drug Deliv. Rev..

[45] Blanco E., Shen H., Ferrari M. (2015). Principles of nanoparticle design for overcoming biological barriers to drug delivery. Nat Biotechnol..

[46] Sriraman S.K., Aryasomayajula B., Torchilin V.P. (2014). Barriers to drug delivery in solid tumors. Tissue Barriers.

[47] Deshmukh S.K., Srivastava S.K., Tyagi N., Ahmad A., Singh A.P., Ghadhban A.A.L., Dyess D.L., Carter J.E., Dugger K., Singh S. (2017). Emerging evidence for the role of differential tumor microenvironment in breast cancer racial disparity: A closer look at the surroundings. Carcinogenesis.

[48] Yu T., Di G. (2017). Role of tumor microenvironment in triple-negative breast cancer and its prognostic significance. Chin. J. Cancer Res..

[49] Soysal S.D., Tzankov A., Muenst S.E. (2015). Role of the Tumor Microenvironment in Breast Cancer. Pathobiology.

[50] Johnson R., Sabnis N., McConathy W.J., Lacko A.G. (2013). The potential role of nanotechnology in therapeutic approaches for triple negative breast cancer. Pharmaceutics.

[51] Zhu X., Zhou W. (2015). The emerging regulation of VEGFR-2 in triple-negative breast cancer. Front. Endocrinol..

[52] Sukumar J., Gast K., Quiroga D., Lustberg M., Williams N. (2021). Triple-negative breast cancer: Promising prognostic biomarkers currently in development. Expert Rev. Anticancer Ther..

[53] Zhao Z., Li Y., Shukla R., Liu H., Jain A., Barve A., Cheng K. (2019). Development of a Biocompatible Copolymer Nanocomplex to Deliver VEGF siRNA for Triple Negative Breast Cancer. Theranostics.

[54] Ghosh S., Javia A., Shetty S., Bardoliwala D., Maiti K., Banerjee S., Khopade A., Misra A., Sawant K., Bhowmick S. (2021). Triple negative breast cancer and non-small cell lung cancer: Clinical challenges and nano-formulation approaches. J. Control Rel..

[55] Nakai K., Hung M.C., Yamaguchi H. (2016). A perspective on anti-EGFR therapies targeting triple-negative breast cancer. Am. J. Cancer Res..

[56] Wang Y., Zhang T., Kwiatkowski N., Abraham B.J., Lee T.I., Xie S., Yuzugullu H., Von T., Li H., Lin Z. (2015). CDK7-Dependent Transcriptional Addiction in Triple-Negative Breast Cancer. Cell.

[57] Si Y., Xu Y., Guan J.S., Chen K., Kim S., Yang E.S., Zhou L., Liu X.M. (2021). Anti-EGFR antibody-drug conjugate for triple-negative breast cancer therapy. Eng. Life Sci..

[58] Su Y.C., Burnouf P.A., Chuang K.H., Chen B.M., Cheng T.L., Roffler S.R. (2017). Conditional internalization of PEGylated nanomedicines by PEG engagers for triple negative breast cancer therapy. Nat. Commun..

[59] Pawar A., Prabhu P. (2019). Biomedicine & Pharmacotherapy Nanosoldiers: A promising strategy to combat triple negative breast cancer. Biomed. Pharmacother..

[60] Geenen J.J.J., Linn S.C., Beijnen J.H., Schellens J.H.M. (2018). PARP Inhibitors in the Treatment of Triple-Negative Breast Cancer. Clin Pharmacokinet..

[61] Lyons T.G. (2019). Targeted Therapies for Triple-Negative Breast Cancer. Curr. Treat. Options Oncol..

[62] Mehta A.K., Cheney E.M., Hartl C.A., Pantelidou C., Oliwa M., Castrillon J.A., Lin J.R., Hurst K.E., de Oliveira Taveira M., Johnson N.T. (2021). Targeting immunosuppressive macrophages overcomes PARP inhibitor resistance in BRCA1-associated triple-negative breast cancer. Nat. Cancer.

[63] Massihnia D., Galvano A., Fanale D., Perez A., Castiglia M., Incorvaia L., Listì A., Rizzo S., Cicero G., Bazan V. (2016). Triple negative breast cancer: Shedding light onto the role of pi3k/akt/mtor pathway. Oncotarget.

[64] Chan J.J., Tan T.J., Dent R. (2019). Novel therapeutic avenues in triple-negative breast cancer: PI3K/AKT inhibition, androgen receptor blockade, and beyond. Ther. Adv. Med. Oncol..

[65] Cretella D., Ravelli A., Fumarola C., La Monica S., Digiacomo G., Cavazzoni A., Alfieri R., Biondi A., Generali D., Bonelli M. (2018). The anti-tumor efficacy of CDK4/6 inhibition is enhanced by the combination with PI3K/AKT/mTOR inhibitors through impairment of glucose metabolism in TNBC cells. J. Exp. Clin. Cancer Res..

[66] Kim K.Y., Park K.I., Kim S.H., Yu S.N., Park S.G., Kim Y.W., Seo Y.K., Ma J.Y., Ahn S.C. (2017). Inhibition of autophagy promotes salinomycin-induced apoptosis via reactive oxygen species-mediated PI3K/AKT/mTOR and ERK/p38 MAPK-dependent signaling in human prostate cancer cells. Int. J. Mol. Sci..

[67] Marra A., Viale G., Curigliano G. (2019). Recent advances in triple negative breast cancer: The immunotherapy era. BMC Medicine.

[68] Stovgaard E.S., Nielsen D., Hogdall E., Balslev E. (2018). Triple negative breast cancer–prognostic role of immune-related factors: A systematic review. Acta Oncol..

[69] Dees S., Ganesan R., Singh S., Grewal I.S. (2021). Bispecific Antibodies for Triple Negative Breast Cancer. Trends Cancer.

[70] Wang Z., Sau S., Alsaab H.O., Iyer A.K. (2018). CD44 directed nanomicellar payload delivery platform for selective anticancer effect and tumor specific imaging of triple negative breast cancer. Nanomed. Nanotechnol. Biol Med..

[71] Lee J., Kim D.M., Lee A. (2019). Prognostic role and clinical association of tumor-infiltrating lymphocyte, programmed death ligand-1 expression with neutrophil-lymphocyte ratio in locally advanced triple-negative breast cancer. Cancer Res. Treat..

[72] Mina L.A., Lim S., Bahadur S.W., Firoz A.T. (2019). Immunotherapy for the treatment of breast cancer: Emerging new data. Breast Cancer Targets Ther..

[73] Singh S., Numan A., Maddiboyina B., Arora S., Riadi Y., Md S., Alhakamy N.A., Kesharwani P. (2021). The emerging role of immune checkpoint inhibitors in the treatment of triple-negative breast cancer. Drug Discov. Today.

[74] Kagihara J.A., Andress M., Diamond J.R. (2020). Nab-paclitaxel and atezolizumab for the treatment of PD-L1-positive, metastatic triple-negative breast cancer: Review and future directions. Expert Rev. Precis. Med. Drug Dev..

[75] Du X., Tang F., Liu M., Su J., Zhang Y., Wu W., Devenport M., Lazarski C.A., Zhang P., Wang X. (2018). A reappraisal of CTLA-4 checkpoint blockade in cancer immunotherapy. Cell Res..

[76] Ahmadzada T., Reid G., McKenzie D.R. (2018). Fundamentals of siRNA and miRNA therapeutics and a review of targeted nanoparticle delivery systems in breast cancer. Biophys. Rev..

[77] Mirza Z., Karim S. (2021). Nanoparticles-based drug delivery and gene therapy for breast cancer: Recent advancements and future challenges. Semin. Cancer Biol..

[78] Liu Y., Zhu Y.H., Mao C.Q., Dou S., Shen S., Tan Z.B., Wang J. (2014). Triple negative breast cancer therapy with CDK1 siRNA delivered by cationic lipid assisted PEG-PLA nanoparticles. J. Control. Rel..

[79] Alshaer W., Hillaireau H., Vergnaud J., Mura S., Deloménie C., Sauvage F., Ismail S., Fattal E. (2018). Aptamer-guided siRNA-loaded nanomedicines for systemic gene silencing in CD-44 expressing murine triple-negative breast cancer model. J. Control. Rel..

[80] Tang J., Howard C.B., Mahler S.M., Thurecht K.J., Huang L., Xu Z.P. (2018). Enhanced delivery of siRNA to triple negative breast cancer cells in vitro and in vivo through functionalizing lipid-coated calcium phosphate nanoparticles with dual target ligands. Nanoscale.

[81] Qattan A. (2020). Novel mirna targets and therapies in the triple-negative breast cancer microenvironment: An emerging hope for a challenging disease. Int. J. Mol. Sci..

[82] Zhu H., Dai M., Chen X., Chen X., Qin S., Dai S. (2017). Integrated analysis of the potential roles of miRNA-mRNA networks in triple negative breast cancer. Mol. Med. Rep..

[83] Bhargava-Shah A., Foygel K., Devulapally R., Paulmurugan R. (2016). Orlistat and antisense-miRNA-loaded PLGA-PEG nanoparticles for enhanced triple negative breast cancer therapy. Nanomedicine.

[84] Dobson J., de Queiroz G.F., Golding J.P. (2018). Photodynamic therapy and diagnosis: Principles and comparative aspects. Vet. J..

[85] Fernandes S.R.G., Fernandes R., Sarmento B., Pereira P.M.R., Tomé J.P.C. (2019). Photoimmunoconjugates: Novel synthetic strategies to target and treat cancer by photodynamic therapy. Org. Biomol. Chem..

[86] Nitheesh Y., Pradhan R., Hejmady S., Taliyan R., Singhvi G., Alexander A., Kesharwani P., Dubey S.K. (2021). Surface engineered nanocarriers for the management of breast cancer. Mater. Sci. Eng. C.

[87] Sivasubramanian M., Chuang Y.C., Lo L.W. (2019). Evolution of nanoparticle-mediated photodynamic therapy: From superficial to deep-seated cancers. Molecules.

[88] Banerjee S.M., Macrobert A.J., Mosse C.A., Periera B., Bown S.G., Keshtgar M.R.S. (2017). Photodynamic therapy: Inception to application in breast cancer. Breast.

[89] Dos Santos A.F., De Almeida D.R.Q., Terra L.F., Baptista M.S., Labriola L. (2019). Photodynamic therapy in cancer treatment–An update review. J. Cancer Metastasis Treat..

[90] Van Straten D., Mashayekhi V., De Bruijn H.S., Oliveira S., Robinson D.J. (2017). Oncologic Photodynamic Therapy: Basic Principles, Current Clinical Status and Future Directions. Cancers.

[91] Kwiatkowski S., Knap B., Przystupski D., Saczko J., Kędzierska E., Knap-Czop K., Kotlińska J., Michel O., Kotowski K., Kulbacka J. (2018). Photodynamic therapy—mechanisms, photosensitizers and combinations. Biomed. Pharmacother..

[92] Castano A.P., Demidova T.N., Hamblin M.R. (2004). Mechanisms in photodynamic therapy: Part one—Photosensitizers, photochemistry and cellular localization. Photodiagnosis Photodyn. Ther..

[93] Castano A.P., Mroz P., Hamblin M.R. (2006). Photodynamic therapy and anti-tumour immunity. Nat. Rev. Cancer.

[94] Shemesh C.S., Hardy C.W., Yu D.S., Fernandez B., Zhang H. (2014). Indocyanine green loaded liposome nanocarriers for photodynamic therapy using human triple negative breast cancer cells. Photodiagnosis Photodyn. Ther..

[95] Sun S., Xu Y., Fu P., Chen M., Sun S., Zhao R., Wang J., Liang X., Wang S. (2018). Ultrasound-targeted photodynamic and gene dual therapy for effectively inhibiting triple negative breast cancer by cationic porphyrin lipid microbubbles loaded with HIF1α-siRNA. Nanoscale.

[96] Zhao L., Zhang X., Wang X., Guan X., Zhang W., Ma J. (2021). Recent advances in selective photothermal therapy of tumor. J. Nanobiotechnol..

[97] Gao G., Jiang Y.W., Guo Y., Jia H.R., Cheng X., Deng Y., Yu X.W., Zhu Y.X., Guo H.Y., Sun W. (2020). Enzyme-Mediated Tumor Starvation and Phototherapy Enhance Mild-Temperature Photothermal Therapy. Adv. Funct. Mater..

[98] Valcourt D.M., Dang M.N., Day E.S. (2019). IR820-loaded PLGA nanoparticles for photothermal therapy of triple-negative breast cancer. J. Biomed. Mater. Res. Part A.

[99] Zhao S., Tian Y., Liu W., Su Y., Zhang Y., Teng Z., Zhao Y., Wang S., Lu G., Yu Z. (2018). High and low molecular weight hyaluronic acid-coated gold nanobipyramids for photothermal therapy. RSC Adv..

[100] Zhang M., Kim H.S., Jin T., Woo J., Piao Y.J., Moon W.K. (2017). Near-infrared photothermal therapy using anti-EGFR-gold nanorod conjugates for triple negative breast cancer. Oncotarget.

[101] Wang S., Tian Y., Tian W., Sun J., Zhao S., Liu Y., Wang C., Tang Y., Ma X., Teng Z. (2016). Selectively Sensitizing Malignant Cells to Photothermal Therapy Using a CD44-Targeting Heat Shock Protein 72 Depletion Nanosystem. ACS Nano.

[102] Tian Y., Lei M. (2019). Polydopamine-Based Composite Nanoparticles with Redox-Labile Polymer Shells for Controlled Drug Release and Enhanced Chemo-Photothermal Therapy. Nanoscale Res. Lett..

[103] Rosenthal I., Sostaric J.Z., Riesz P. (2004). Sonodynamic therapya review of the synergistic effects of drugs and ultrasound. Ultrason. Sonochem..

[104] Carovac A., Smajlovic F., Junuzovic D. (2011). Application of Ultrasound in Medicine. Acta Inf. Med..

[105] Han X., Song Z., Zhou Y., Zhang Y., Deng Y., Qin J., Zhang T., Jiang Z. (2021). Mitochondria-targeted high-load sound-sensitive micelles for sonodynamic therapy to treat triple-negative breast cancer and inhibit metastasis. Mater. Sci. Eng C.

[106] Li Y.S., Reid C.N., McHale A.P. (2008). Enhancing ultrasound-mediated cell membrane permeabilisation (sonoporation) using a high frequency pulse regime and implications for ultrasound-aided cancer chemotherapy. Cancer Lett..

[107] Nomikou N., Li Y.S., McHale A.P. (2010). Ultrasound-enhanced drug dispersion through solid tumours and its possible role in aiding ultrasound-targeted cancer chemotherapy. Cancer Lett..

[108] Feng X., Wu C., Yang W., Wu J., Wang P. (2022). Mechanism-Based Sonodynamic–Chemo Combinations against Triple-Negative Breast Cancer. Int. J. Mol. Sci..

[109] Chen H., Liu L., Ma A., Yin T., Chen Z., Liang R., Qiu Y., Zheng M., Cai L. (2021). Noninvasively immunogenic sonodynamic therapy with manganese protoporphyrin liposomes against triple-negative breast cancer. Biomaterials.

[110] Thakur V., Kutty R.V. (2019). Recent advances in nanotheranostics for triple negative breast cancer treatment. J. Exp. Clin. Cancer Res..

[111] El-Sahli S., Hua K., Sulaiman A., Chambers J., Li L., Farah E., McGarry S., Liu D., Zheng P., Lee S.-H. (2021). A triple-drug nanotherapy to target breast cancer cells, cancer stem cells, and tumor vasculature. Cell Death Dis..

[112] Mukherjee A., Waters A.K., Kalyan P., Achrol A.S., Kesari S., Yenugonda V.M. (2019). Lipid-polymer hybrid nanoparticles as a next-generation drug delivery plkatform: State of the art, emerging technologies, and perspectives. Int. J. Nanomed..

[113] Tran S., DeGiovanni P., Piel B., Rai P. (2017). Cancer nanomedicine: A review of recent success in drug delivery. Clin. Transl. Med..

[114] Teles R.H.G., Moralles H.F., Cominetti M.R. (2018). Global trends in nanomedicine research on triple negative breast cancer: A bibliometric analysis. Int. J. Nanomed..

[115] Saeed N.A., Hamzah I.H., Mahmood S.I. (2021). The applications of nano-medicine in the breast cancer therapy. J. Phys. Conf. Ser..

[116] Fang F., Li M., Zhang J., Lee C.S. (2020). Different Strategies for Organic Nanoparticle Preparation in Biomedicine. ACS Mater. Lett..

[117] Romero G., Moya S.E. (2012). Synthesis of organic nanoparticles. Front. Nanosci..

[118] Mitragotri S., Stayton P. (2014). Organic nanoparticles for drug delivery and imaging. MRS Bull..

[119] Chaudhuri A., Kumar D.N., Shaik R.A., Eid B.G., Abdel-Naim A.B., Md S., Ahmad A., Agrawal A.K. (2022). Lipid-Based Nanoparticles as a Pivotal Delivery Approach in Triple Negative Breast Cancer (TNBC) Therapy. Int. J. Mol. Sci..

[120] Shi J., Kantoff P.W., Wooster R., Farokhzad O.C. (2017). Cancer nanomedicine: Progress, challenges and opportunities. Nat. Rev. Cancer.

[121] Bourquin J., Milosevic A., Hauser D., Lehner R., Blank F., Petri-Fink A., Rothen-Rutishauser B. (2018). Biodistribution, Clearance, and Long-Term Fate of Clinically Relevant Nanomaterials. Adv. Mater..

[122] Bozzuto G., Molinari A. (2015). Liposomes as nanomedical devices. Int. J. Nanomed..

[123] Zamani P., Momtazi-Borojeni A.A., Nik M.E., Oskuee R.K., Sahebkar A. (2018). Nanoliposomes as the adjuvant delivery systems in cancer immunotherapy. J. Cell Physiol..

[124] Gonda A., Zhao N., Shah J.V., Calvelli H.R., Kantamneni H., Francis N.L., Ganapathy V. (2019). Engineering Tumor-Targeting Nanoparticles as Vehicles for Precision Nanomedicine. Med. One.

[125] Beltrán-Gracia E., López-Camacho A., Higuera-Ciapara I., Velázquez-Fernández J.B., Vallejo-Cardona A.A. (2019). Nanomedicine review: Clinical developments in liposomal applications. Cancer Nanotechnol..

[126] Immordino M.L., Dosio F., Cattel L. (2006). Stealth liposomes: Review of the basic science, rationale, and clinical applications, existing and potential. Int. J. Nanomed..

[127] Si Y., Zhang Y., Ngo H.G., Guan J.-S., Chen K., Wang Q., Singh A.P., Xu Y., Zhou L., Yang E.S. (2021). Targeted Liposomal Chemotherapies to Treat Triple-Negative Breast Cancer. Cancers.

[128] Maji I., Mahajan S., Sriram A., Medtiya P., Vasave R., Khatri D.K., Kumar R., Singh S.B., Madan J., Singh P.K. (2021). Solid self emulsifying drug delivery system: Superior mode for oral delivery of hydrophobic cargos. J. Control. Release.

[129] Gursoy R.N., Benita S. (2004). Self-emulsifying drug delivery systems (SEDDS) for improved oral delivery of lipophilic drugs. Biomed Pharmacother..

[130] Valicherla G.R., Dave K.M., Syed A.A., Riyazuddin M., Gupta A.P., Singh A., Wahajuddin, Mitra K., Datta D., Gayen J.R. (2016). Formulation optimization of Docetaxel loaded self-emulsifying drug delivery system to enhance bioavailability and anti-tumor activity. Sci. Rep..

[131] Shrivastava N., Parikh A., Dewangan R.P., Biswas L., Verma A.K., Mittal S., Ali J., Garg S., Baboota S. (2022). Solid Self-Nano Emulsifying Nanoplatform Loaded with Tamoxifen and Resveratrol for Treatment of Breast Cancer. Pharmaceutics.

[132] Timur S.S., Yöyen-Ermiş D., Esendağlı G., Yonat S., Horzum U., Esendağlı G., Gürsoy R.N. (2019). Efficacy of a novel LyP-1-containing self-microemulsifying drug delivery system (SMEDDS) for active targeting to breast cancer. Eur. J. Pharm. Biopharm..

[133] Scioli Montoto S., Muraca G., Ruiz M.E. (2020). Solid Lipid Nanoparticles for Drug Delivery: Pharmacological and Biopharmaceutical Aspects. Front. Mol. Biosci..

[134] Kothari I.R., Mazumdar S., Sharma S., Italiya K., Mittal A., Chitkara D. (2019). Docetaxel and alpha-lipoic acid co-loaded nanoparticles for cancer therapy. Ther. Deliv..

[135] Ss Pindiprolu S.K., Krishnamurthy P.T., Ghanta V.R., Chintamaneni P.K. (2020). Phenyl boronic acid-modified lipid nanocarriers of niclosamide for targeting triple-negative breast cancer. Nanomedicine.

[136] Garg J., Pathania K., Sah S.P., Pawar S.V. (2022). Nanostructured lipid carriers: A promising drug carrier for targeting brain tumours. Futur. J. Pharm. Sci..

[137] Haider M., Abdin S.M., Kamal L., Orive G. (2020). Nanostructured lipid carriers for delivery of chemotherapeutics: A review. Pharmaceutics.

[138] Andey T., Sudhakar G., Marepally S., Patel A., Banerjee R., Singh M. (2015). Lipid nanocarriers of a lipid-conjugated estrogenic derivative inhibit tumor growth and enhance Cisplatin activity against triple-negative breast cancer: Pharmacokinetic and efficacy evaluation. Mol. Pharm..

[139] Kutty R.V., Feng S.-S. (2013). Cetuximab conjugated vitamin E TPGS micelles for targeted delivery of docetaxel for treatment of triple negative breast cancers. Biomaterials.

[140] Selestin Raja I., Thangam R., Fathima N.N. (2018). Polymeric Micelle of a Gelatin-Oleylamine Conjugate: A Prominent Drug Delivery Carrier for Treating Triple Negative Breast Cancer Cells. ACS Appl. Bio. Mater..

[141] Wang Y., Wang Y., Chen G., Li Y., Xu W., Gong S. (2017). Quantum-Dot-Based Theranostic Micelles Conjugated with an Anti-EGFR Nanobody for Triple-Negative Breast Cancer Therapy. ACS Appl. Mater. Interfaces.

[142] Zhang Y., Zhu X., Chen X., Chen Q., Zhou W., Guo Q., Lu Y., Li C., Zhang Y., Liang D. (2019). Activated Platelets-Targeting Micelles with Controlled Drug Release for Effective Treatment of Primary and Metastatic Triple Negative Breast Cancer. Adv. Funct. Mater..

[143] Dubey S.K., Salunkhe S., Agrawal M., Kali M., Singhvi G., Tiwari S., Saraf S., Saraf S., Alexander A. (2020). Understanding the Pharmaceutical Aspects of Dendrimers for the Delivery of Anticancer Drugs. Curr. Drug. Targets.

[144] Jang W.-D., Selim K., Lee C.-H., Kang I.-K. (2009). Bioinspired application of dendrimers: From bio-mimicry to biomedical applications. Prog. Polym. Sci..

[145] Lin Q., Jiang G., Tong K. (2012). Dendrimers in Drug-Delivery Applications. Des. Monomers Polym..

[146] Dubey S.K., Kali M., Hejmady S., Saha R.N., Alexander A., Kesharwani P. (2021). Recent advances of dendrimers as multifunctional nano-carriers to combat breast cancer. Eur. J. Pharm. Sci. Off J. Eur. Fed. Pharm. Sci..

[147] Ghosh S., Ghosal K., Mohammad S.A., Sarkar K. (2019). Dendrimer functionalized carbon quantum dot for selective detection of breast cancer and gene therapy. Chem. Eng. J..

[148] Jain A., Mahira S., Majoral J.-P., Bryszewska M., Khan W., Ionov M. (2019). Dendrimer mediated targeting of siRNA against polo-like kinase for the treatment of triple negative breast cancer. J. Biomed. Mater Res. A.

[149] Liu C., Gao H., Zhao Z., Rostami I., Wang C., Zhu L., Yang Y. (2019). Improved tumor targeting and penetration by a dual-functional poly(amidoamine) dendrimer for the therapy of triple-negative breast cancer. J. Mater. Chem. B..

[150] Finlay J., Roberts C.M., Lowe G., Loeza J., Rossi J.J., Glackin C.A. (2015). RNA-based TWIST1 inhibition via dendrimer complex to reduce breast cancer cell metastasis. BioMed Res Int..

[151] Surekha B., Kommana N.S., Dubey S.K., Kumar A.V.P., Shukla R., Kesharwani P. (2021). PAMAM dendrimer as a talented multifunctional biomimetic nanocarrier for cancer diagnosis and therapy. Colloids Surf. B Biointerfaces.

[152] Mogoşanu G.D., Grumezescu A.M., Bejenaru C., Bejenaru L.E. (2016). Polymeric protective agents for nanoparticles in drug delivery and targeting. Int. J. Pharm..

[153] van Vlerken L.E., Vyas T.K., Amiji M.M. (2007). Poly(ethylene glycol)-modified Nanocarriers for Tumor-targeted and Intracellular Delivery. Pharm. Res..

[154] Li B., Li Q., Mo J., Dai H. (2017). Drug-Loaded Polymeric Nanoparticles for Cancer Stem Cell Targeting. Front. Pharmacol..

[155] Fortuni B., Inose T., Ricci M., Fujita Y., Van Zundert I., Masuhara A., Fron E., Mizuno H., Latterini L., Rocha S. (2019). Polymeric Engineering of Nanoparticles for Highly Efficient Multifunctional Drug Delivery Systems. Sci. Rep..

[156] Mamnoon B., Loganathan J., Confeld M.I., De Fonseka N., Feng L., Froberg J., Choi Y., Tuvin D.M., Sathish V., Mallik S. (2021). Targeted polymeric nanoparticles for drug delivery to hypoxic, triple-negative breast tumors. ACS Appl. Bio. Mater..

[157] Zhou Y., Chen D., Xue G., Yu S., Yuan C., Huang M., Jiang L. (2020). Improved therapeutic efficacy of quercetin-loaded polymeric nanoparticles on triple-negative breast cancer by inhibiting uPA. RSC Adv..

[158] Devulapally R., Sekar N.M., Sekar T.V., Foygel K., Massoud T.F., Willmann J.K., Paulmurugan R. (2015). Polymer nanoparticles mediated codelivery of antimiR-10b and antimiR-21 for achieving triple negative breast cancer therapy. ACS Nano.

[159] Deng Z.J., Morton S.W., Ben-Akiva E., Dreaden E.C., Shopsowitz K.E., Hammond P.T. (2013). Layer-by-layer nanoparticles for systemic codelivery of an anticancer drug and siRNA for potential triple-negative breast cancer treatment. ACS Nano.

[160] Shi P., Aluri S., Lin Y.-A., Shah M., Edman M., Dhandhukia J., Cui H., MacKay J.A. (2013). Elastin-based protein polymer nanoparticles carrying drug at both corona and core suppress tumor growth in vivo. J. Control Release.

[161] Jain V., Kumar H., Anod H.V., Chand P., Gupta N.V., Dey S., Kesharwani S.S. (2020). A review of nanotechnology-based approaches for breast cancer and triple-negative breast cancer. J. Control Release.

[162] Koleva L., Bovt E., Ataullakhanov F., Sinauridze E. (2020). Erythrocytes as carriers: From drug delivery to biosensors. Pharmaceutics.

[163] Wang C., Wang M., Zhang Y., Jia H., Chen B. (2022). Cyclic arginine-glycine-aspartic acid-modified red blood cells for drug delivery: Synthesis and in vitro evaluation. J. Pharm. Anal..

[164] Chen Y., Zhang Y. (2018). Application of the CRISPR/Cas9 System to Drug Resistance in Breast Cancer. Adv. Sci..

[165] Farheen J., Hosmane N.S., Zhao R., Zhao Q., Iqbal M.Z., Kong X. (2022). Nanomaterial-assisted CRISPR gene-engineering—A hallmark for triple-negative breast cancer therapeutics advancement. Mater Today Bio..

[166] Zhang L., Wang P., Feng Q., Wang N., Chen Z., Huang Y., Zheng W., Jiang X. (2017). Lipid nanoparticle-mediated efficient delivery of CRISPR/Cas9 for tumor therapy. NPG Asia Mater..

[167] Tian Y., Li S., Song J., Ji T., Zhu M., Anderson G.J., Wei J., Nie G. (2014). A doxorubicin delivery platform using engineered natural membrane vesicle exosomes for targeted tumor therapy. Biomaterials.

[168] Singh R., Pochampally R., Watabe K., Lu Z., Mo Y.Y. (2014). Exosome-mediated transfer of miR-10b promotes cell invasion in breast cancer. Mol. Cancer.

[169] Prodana M., Ionita D., Ungureanu C., Bojin D., Demetrescu I. (2011). Enhancing Antibacterial Effect of Multiwalled Carbon Nanotubes using Silver Nanoparticles. Dig. J. Nanomater Biostruct..

[170] Badea M.A., Prodana M., Dinischiotu A., Crihana C., Ionita D., Balas M. (2018). Cisplatin Loaded Multiwalled Carbon Nanotubes Induce Resistance in Triple Negative Breast Cancer Cells. Pharmaceutics.

[171] Fahrenholtz C., Ding S., Bernish B., Wright M., Bierbach U., Singh R. (2016). Abstract B05: Self-assembling platinum-acridine loaded carbon nanotubes for triple-negative breast cancer chemotherapy. Mol. Cancer Res..

[172] Singhai N.J., Maheshwari R., Ramteke S. (2020). CD44 receptor targeted ‘smart’ multi-walled carbon nanotubes for synergistic therapy of triple-negative breast cancer. Colloid Interface Sci. Commun..

[173] Kim D., Kim J., Park Y.I., Lee N., Hyeon T. (2018). Recent Development of Inorganic Nanoparticles for Biomedical Imaging. ACS Cent Sci..

[174] Anselmo A.C., Mitragotri S. (2015). A Review of Clinical Translation of Inorganic Nanoparticles. AAPS J..

[175] Croissant J.G., Butler K.S., Zink J.I., Brinker C.J. (2020). Synthetic amorphous silica nanoparticles: Toxicity, biomedical and environmental implications. Nat. Rev. Mater..

[176] Cheng Y., Chen Q., Guo Z., Li M., Yang X., Wan G., Chen H., Zhang Q., Wang Y. (2020). An Intelligent Biomimetic Nanoplatform for Holistic Treatment of Metastatic Triple-Negative Breast Cancer via Photothermal Ablation and Immune Remodeling. ACS Nano.

[177] Zhang T., Liu H., Li L., Guo Z., Song J., Yang X., Wan G., Li R., Wang Y. (2021). Leukocyte/platelet hybrid membrane-camouflaged dendritic large pore mesoporous silica nanoparticles co-loaded with photo/chemotherapeutic agents for triple negative breast cancer combination treatment. Bioact. Mater..

[178] Wu X., Han Z., Schur R.M., Lu Z.-R. (2016). Targeted Mesoporous Silica Nanoparticles Delivering Arsenic Trioxide with Environment Sensitive Drug Release for Effective Treatment of Triple Negative Breast Cancer. ACS Biomater. Sci. Eng..

[179] Siddiqi K.S., Husen A., Rao R.A.K. (2018). A review on biosynthesis of silver nanoparticles and their biocidal properties. J. Nanobiotechnol..

[180] Lee S.H., Jun B.-H. (2019). Silver Nanoparticles: Synthesis and Application for Nanomedicine. Int. J. Mol. Sci..

[181] Azizi M., Ghourchian H., Yazdian F., Bagherifam S., Bekhradnia S., Nyström B. (2017). Anti-cancerous effect of albumin coated silver nanoparticles on MDA-MB 231 human breast cancer cell line. Sci. Rep..

[182] Swanner J., Fahrenholtz C., Tenvooren I., Bernish B., Sears J., Hooker A., Furdui C., Alli E., Li W., Donati G. (2019). Silver nanoparticles selectively treat triple negative breast cancer cells without affecting non-malignant breast epithelial cells in vitro and in vivo. FASEB BioAdv..

[183] Sears J.J. (2018). Nanoparticle Based Multi-Modal Therapies Against Triple Negative Breast Cancer. Ph.D. Thesis.

[184] Surapaneni S.K., Bashir S., Tikoo K. (2018). Gold nanoparticles-induced cytotoxicity in triple negative breast cancer involves different epigenetic alterations depending upon the surface charge. Sci. Rep..

[185] Castilho M.L., Jesus V.P.S., Vieira P.F.A., Hewitt K.C., Raniero L. (2021). Chlorin e6-EGF conjugated gold nanoparticles as a nanomedicine based therapeutic agent for triple negative breast cancer. Photodiagn. Photodyn Ther..

[186] Madni A., Tahir N., Rehman M., Raza A., Mahmood M.A., Khan M.I., Kashif P.M. (2017). Hybrid Nano-carriers for Potential Drug Delivery. Adv. Technol. Deliv. Ther..

[187] Xia T., Kovochich M., Liong M., Meng H., Kabehie S., George J.I., Zink S., Nel A.E. (2009). Polyethyleneimine Coating Enhances the Cellular Uptake of Mesoporous Silica Nanoparticles and Allows Safe Delivery of siRNA and DNA Constructs. ACS Nano.

[188] Ahir M., Upadhyay P., Ghosh A., Sarker S., Bhattacharya S., Gupta P., Ghosh S., Chattopadhyay S., Adhikary A. (2020). Delivery of dual miRNA through CD44-targeted mesoporous silica nanoparticles for enhanced and effective triple-negative breast cancer therapy. Biomater. Sci..

[189] Laha D., Pal K., Chowdhuri A.R., Parida P.K., Sahu S.K., Jana K., Karmakar P. (2019). Fabrication of curcumin-loaded folic acid-tagged metal organic framework for triple negative breast cancer therapy in in vitro and in vivo systems. New J. Chem..

[190] Bhardwaj A., Kumar L., Mehta S., Mehta A. (2015). Stimuli-sensitive systems—An emerging delivery system for drugs. Artif. Cells Nanomed. Biotechnol..

[191] Yu J., Chu X., Hou Y. (2014). Stimuli-responsive cancer therapy based on nanoparticles. Chem. Commun..

[192] Nakayama M., Okano T., Miyazaki T., Kohori F., Sakai K., Yokoyama M. (2006). Molecular design of biodegradable polymeric micelles for temperature-responsive drug release. J. Control Release.

[193] Ganta S., Devalapally H., Shahiwala A., Amiji M. (2008). A review of stimuli-responsive nanocarriers for drug and gene delivery. J. Control Release.

[194] Fleige E., Quadir M.A., Haag R. (2012). Stimuli-responsive polymeric nanocarriers for the controlled transport of active compounds: Concepts and applications. Adv. Drug Deliv. Rev..

[195] Cheng R., Meng F., Deng C., Klok H.-A., Zhong Z. (2013). Dual and multi-stimuli responsive polymeric nanoparticles for programmed site-specific drug delivery. Biomaterials.

[196] Torchilin V.P. (2014). Multifunctional, stimuli-sensitive nanoparticulate systems for drug delivery. Nat. Rev. Drug Discov..

[197] Patil R., Portilla-Arias J., Ding H., Konda B., Rekechenetskiy A., Inoue S., Black K.L., Holler E., Ljubimova J.Y. (2012). Cellular delivery of doxorubicin via pH-controlled hydrazone linkage using multifunctional nano vehicle based on poly(β-l-malic acid). Int. J. Mol. Sci..

[198] Cimen Z., Babadag S., Odabas S., Altuntas S., Demirel G., Demirel G.B. (2021). Injectable and Self-Healable pH-Responsive Gelatin–PEG/Laponite Hybrid Hydrogels as Long-Acting Implants for Local Cancer Treatment. ACS Appl. Polym. Mater..

[199] Hwang J.-H., Choi C.W., Kim H.-W., Kim D.H., Kwak T.W., Lee H.M., hyun Kim C., Chung C.W., Jeong Y.-I., Kang D.H. (2013). Dextran-b-poly (L-histidine) copolymer nanoparticles for pH-responsive drug delivery to tumor cells. Int. J. Nanomed..

[200] Min K.H., Kim J.-H., Bae S.M., Shin H., Kim M.S., Park S., Lee H., Park R.-W., Kim I.-S., Kim K. (2010). Tumoral acidic pH-responsive MPEG-poly (β-amino ester) polymeric micelles for cancer targeting therapy. J. Control. Release.

[201] Niu S., Williams G.R., Wu J., Wu J., Zhang X., Chen X., Li S., Jiao J., Zhu L.-M. (2019). A chitosan-based cascade-responsive drug delivery system for triple-negative breast cancer therapy. J. Nanobiotechnol..

[202] He X., Zhang J., Li C., Zhang Y., Lu Y., Zhang Y., Liu L., Ruan C., Chen Q., Chen X. (2018). Enhanced bioreduction-responsive diselenide-based dimeric prodrug nanoparticles for triple negative breast cancer therapy. Theranostics.

[203] Zhang J., Zuo T., Liang X., Xu Y., Yang Y., Fang T., Li J., Chen D., Shen Q. (2019). Fenton-reaction-stimulative nanoparticles decorated with a reactive-oxygen-species (ROS)-responsive molecular switch for ROS amplification and triple negative breast cancer therapy. J. Mater. Chem. B.

[204] Radhakrishnan K., Tripathy J., Gnanadhas D.P., Chakravortty D., Raichur A.M. (2014). Dual enzyme responsive and targeted nanocapsules for intracellular delivery of anticancer agents. RSC Adv..

[205] Wang Y., Li B., Xu F., Han Z., Wei D., Jia D., Zhou Y. (2018). Tough Magnetic Chitosan Hydrogel Nanocomposites for Remotely Stimulated Drug Release. Biomacromolecules.

[206] Thirunavukkarasu G.K., Cherukula K., Lee H., Jeong Y.Y., Park I.-K., Lee J.Y. (2018). Magnetic field-inducible drug-eluting nanoparticles for image-guided thermo-chemotherapy. Biomaterials.

[207] Xie C., Li P., Han L., Wang Z., Zhou T., Deng W., Wang K., Lu X. (2017). Electroresponsive and cell-affinitive polydopamine/polypyrrole composite microcapsules with a dual-function of on-demand drug delivery and cell stimulation for electrical therapy. NPG Asia Mater..

[208] Xin Y., Qi Q., Mao Z., Zhan X. (2017). PLGA nanoparticles introduction into mitoxantrone-loaded ultrasound-responsive liposomes: In vitro and in vivo investigations. Int. J. Pharm..

[209] Paris J.L., Manzano M., Cabañas M.V., Vallet-Regí M. (2018). Mesoporous silica nanoparticles engineered for ultrasound-induced uptake by cancer cells. Nanoscale.

[210] Schmaljohann D. (2006). Thermo- and pH-responsive polymers in drug delivery. Adv. Drug Deliv. Rev..

[211] Liu Y., Wang W., Yang J., Zhou C., Sun J. (2013). pH-sensitive polymeric micelles triggered drug release for extracellular and intracellular drug targeting delivery. Asian J. Pharm. Sci..

[212] Gao W., Chan J.M., Farokhzad O.C. (2010). pH-responsive nanoparticles for drug delivery. Mol. Pharm..

[213] Dutz S., Hergt R. (2013). Magnetic nanoparticle heating and heat transfer on a microscale: Basic principles, realities and physical limitations of hyperthermia for tumour therapy. Int. J. Hyperth. Off. J. Eur. Soc. Hyperth. Oncol. N. Am. Hyperth. Gr..

[214] Ortega D., Pankhurst Q.A. (2013). Magnetic hyperthermia. Nanoscience: Volume 1: Nanostructures through Chemistry.

[215] Anyarambhatla G.R., Needham D. (1999). Enhancement of the Phase Transition Permeability of DPPC Liposomes by Incorporation of MPPC: A New Temperature-Sensitive Liposome for use with Mild Hyperthermia. J. Liposome Res..

[216] Needham D., Anyarambhatla G., Kong G., Dewhirst M.W. (2000). A new temperature-sensitive liposome for use with mild hyperthermia: Characterization and testing in a human tumor xenograft model. Cancer Res..

[217] Li R., Wu W., Liu Q., Wu P., Xie L., Zhu Z., Yang M., Qian X., Ding Y., Yu L. (2013). Intelligently targeted drug delivery and enhanced antitumor effect by gelatinase-responsive nanoparticles. PLoS ONE.

[218] Torchilin V.P. (2018). Fundamentals of Stimuli-responsive Drug and Gene Delivery Systems. Stimuli-Responsive Drug Delivery Systems.

[219] Dubey S.K., Bhatt T., Agrawal M., Saha R.N., Saraf S., Saraf S., Alexander A. (2022). Application of chitosan modified nanocarriers in breast cancer. Int. J. Biol. Macromol..

[220] Ou Y.-C., Webb J., Faley S., Shae D., Talbert E., Lin S., Cutright C., Wilson J., Bellan L., Bardhan R. (2016). Gold Nanoantenna-Mediated Photothermal Drug Delivery from Thermosensitive Liposomes in Breast Cancer. ACS Omega.

[221] Tapeinos C., Pandit A. (2016). Physical, Chemical, and Biological Structures based on ROS-Sensitive Moieties that are Able to Respond to Oxidative Microenvironments. Adv. Mater..

[222] Saravanakumar G., Kim J., Kim W.J. (2017). Reactive-Oxygen-Species-Responsive Drug Delivery Systems: Promises and Challenges. Adv. Sci..

[223] Liu J., Li Y., Chen S., Lin Y., Lai H., Chen B., Chen T. (2020). Biomedical Application of Reactive Oxygen Species-Responsive Nanocarriers in Cancer, Inflammation, and Neurodegenerative Diseases. Front. Chem..

[224] Rasheed T., Bilal M., Abu-Thabit N.Y., Iqbal H.M. (2018). Stimuli Responsive Polymeric Nanocarriers for Drug Delivery Applications.

[225] Basel M.T., Shrestha T.B., Troyer D.L., Bossmann S.H. (2011). Protease-sensitive, polymer-caged liposomes: A method for making highly targeted liposomes using triggered release. ACS Nano.

[226] Liu C., Zhao Z., Gao R., Zhang X., Sun Y., Wu J., Liu J., Chen C. (2022). Matrix Metalloproteinase-2-Responsive Surface-Changeable Liposomes Decorated by Multifunctional Peptides to Overcome the Drug Resistance of Triple-Negative Breast Cancer through Enhanced Targeting and Penetrability. ACS Biomater. Sci. Eng..

[227] Wang Y., Kohane D.S. (2017). External triggering and triggered targeting strategies for drug delivery. Nat. Rev. Mater..

[228] Yang H.Y., Li Y., Lee D.S. (2018). Multifunctional and Stimuli-Responsive Magnetic Nanoparticle-Based Delivery Systems for Biomedical Applications. Adv. Ther..

[229] Schleich N., Danhier F., Préat V. (2015). Iron oxide-loaded nanotheranostics: Major obstacles to in vivo studies and clinical translation. J. Control. Release.

[230] Zhou X., Wang L., Xu Y., Du W., Cai X., Wang F., Ling Y., Chen H., Wang Z., Hu B. (2018). A pH and magnetic dual-response hydrogel for synergistic chemo-magnetic hyperthermia tumor therapy. RSC Adv..

[231] Jeon G., Yang S.Y., Byun J., Kim J.K. (2011). Electrically actuatable smart nanoporous membrane for pulsatile drug release. Nano Lett..

[232] Servant A., Bussy C., Al-Jamal K., Kostarelos K. (2013). Design, engineering and structural integrity of electro-responsive carbon nanotube-based hydrogels for pulsatile drug release. J. Mater Chem. B.

[233] Ge J., Neofytou E., Cahill T.J., Beygui R.E., Zare R.N. (2012). Drug release from electric-field-responsive nanoparticles. ACS Nano.

[234] Hosseini-Nassab N., Samanta D., Abdolazimi Y., Annes J.P., Zare R.N. (2017). Electrically controlled release of insulin using polypyrrole nanoparticles. Nanoscale.

[235] Paris J.L., Cabañas M.V., Manzano M., Vallet-Regí M. (2015). Polymer-Grafted Mesoporous Silica Nanoparticles as Ultrasound-Responsive Drug Carriers. ACS Nano.

[236] Luo Z., Jin K., Pang Q., Shen S., Yan Z., Jiang T., Zhu X., Yu L., Pang Z., Jiang X. (2017). On-Demand Drug Release from Dual-Targeting Small Nanoparticles Triggered by High-Intensity Focused Ultrasound Enhanced Glioblastoma-Targeting Therapy. ACS Appl. Mater. Interfaces.

[237] Raza A., Rasheed T., Nabeel F., Hayat U., Bilal M., Iqbal H.M.N. (2019). Endogenous and Exogenous Stimuli-Responsive Drug Delivery Systems for Programmed Site-Specific Release. Molecules.

[238] Pham S.H., Choi Y., Choi J. (2020). Stimuli-responsive nanomaterials for application in antitumor therapy and drug delivery. Pharmaceutics.

[239] Liu J., Ai X., Cabral H., Liu J., Huang Y., Mi P. (2021). Tumor hypoxia-activated combinatorial nanomedicine triggers systemic antitumor immunity to effectively eradicate advanced breast cancer. Biomaterials.

[240] Zhang R., Li Y., Zhang M., Tang Q., Zhang X. (2016). Hypoxia-responsive drug–drug conjugated nanoparticles for breast cancer synergistic therapy. RSC Adv..

[241] Gurpreet K., Singh S.K. (2018). Review of nanoemulsion formulation and characterization techniques. Indian J. Pharm. Sci..

[242] Di J., Gao X., Du Y., Zhang H., Gao J., Zheng A. (2021). Size, shape, charge and “stealthy” surface: Carrier properties affect the drug circulation time in vivo. Asian J. Pharm Sci..

[243] Patra J.K., Das G., Fraceto L.F., Vangelie E., Campos R., Rodriguez P., Susana L., Torres A., Armando L., Torres D. (2018). Nano based drug delivery systems: Recent developments and future prospects. J. Nanobiotechnol..

[244] Hejmady S., Singhvi G., Saha R.N., Dubey S.K. (2020). Regulatory aspects in process development and scale-up of nanopharmaceuticals. Ther. Deliv..

[245] Ajdary M., Moosavi M.A., Rahmati M., Falahati M., Mahboubi M., Mandegary A., Jangjoo S., Mohammadinejad R., Varma R.S. (2018). Health concerns of various nanoparticles: A review of their in vitro and in vivo toxicity. Nanomaterials.

[246] Hua S., de Matos M.B.C., Metselaar J.M., Storm G. (2018). Current trends and challenges in the clinical translation of nanoparticulate nanomedicines: Pathways for translational development and commercialization. Front. Pharmacol..

